# A comprehensive examination of mental health in patients with head and neck cancer: systematic review and meta-analysis

**DOI:** 10.1093/jncics/pkae031

**Published:** 2024-05-03

**Authors:** Pablo Jimenez-Labaig, Claudia Aymerich, Irene Braña, Antonio Rullan, Jon Cacicedo, Miguel Ángel González-Torres, Kevin J Harrington, Ana Catalan

**Affiliations:** Head and Neck Unit, The Royal Marsden NHS Foundation Trust, London, UK; The Institute of Cancer Research, National Institute of Health Research Biomedical Research Centre, London, UK; Psychiatry Department, Basurto University Hospital. Osakidetza, Basque Health Service, Bilbao, Spain; Biobizkaia Health Research Institute, OSI Bilbao-Basurto, Bilbao, Spain; Centro de Investigación en Red de Salud Mental (CIBERSAM), Madrid, Spain; Lung and Head & Neck Tumors Unit. Medical Oncology Department, Vall d‘Hebron University Hospital, Barcelona, Spain; Vall d’Hebron Institute of Oncology (VHIO), Barcelona, Spain; Head and Neck Unit, The Royal Marsden NHS Foundation Trust, London, UK; The Institute of Cancer Research, National Institute of Health Research Biomedical Research Centre, London, UK; Radiotherapy Department, Cruces University Hospital, Barakaldo, Spain; Biobizkaia Health Research Institute, OSI Ezkerraldea Enkarterri Cruces, Barakaldo, Spain; Faculty of Medicine, University of the Basque Country, Leioa, Spain; Psychiatry Department, Basurto University Hospital. Osakidetza, Basque Health Service, Bilbao, Spain; Biobizkaia Health Research Institute, OSI Bilbao-Basurto, Bilbao, Spain; Centro de Investigación en Red de Salud Mental (CIBERSAM), Madrid, Spain; Neuroscience Department, University of the Basque Country (UPV/EHU), Leioa, Spain; Head and Neck Unit, The Royal Marsden NHS Foundation Trust, London, UK; The Institute of Cancer Research, National Institute of Health Research Biomedical Research Centre, London, UK; Psychiatry Department, Basurto University Hospital. Osakidetza, Basque Health Service, Bilbao, Spain; Biobizkaia Health Research Institute, OSI Bilbao-Basurto, Bilbao, Spain; Centro de Investigación en Red de Salud Mental (CIBERSAM), Madrid, Spain; Neuroscience Department, University of the Basque Country (UPV/EHU), Leioa, Spain; Department of Psychiatry, University of Oxford, Oxford, UK

## Abstract

**Background:**

Patients with head and neck cancer present particularly considerable levels of emotional distress. However, the actual rates of clinically relevant mental health symptoms and disorders among this population remain unknown.

**Methods:**

A Preferred Reporting Items for Systematic Review and Meta-Analyses and Meta-analyses of Observational Studies in Epidemiology–compliant systematic review and quantitative random-effects meta-analysis was performed to determine suicide incidence and the prevalence of depression, anxiety, distress, posttraumatic stress, and insomnia in this population. MEDLINE, Web of Science, Cochrane Central Register, KCI Korean Journal database, SciELO, Russian Science Citation Index, and Ovid-PsycINFO databases were searched from database inception to August 1, 2023 (PROSPERO: CRD42023441432). Subgroup analyses and meta-regressions were performed to investigate the effect of clinical, therapeutical, and methodological factors.

**Results:**

A total of 208 studies (n = 654 413; median age = 60.7 years; 25.5% women) were identified. Among the patients, 19.5% reported depressive symptoms (95% confidence interval [CI] = 17% to 21%), 17.8% anxiety symptoms (95% CI = 14% to 21%), 34.3% distress (95% CI = 29% to 39%), 17.7% posttraumatic symptoms (95% CI = 6% to 41%), and 43.8% insomnia symptoms (95% CI = 35% to 52%). Diagnostic criteria assessments revealed lower prevalence of disorders: 10.3% depression (95% CI = 7% to 13%), 5.6% anxiety (95% CI = 2% to 10%), 9.6% insomnia (95% CI = 1% to 40%), and 1% posttraumatic stress (95% CI = 0% to 84.5%). Suicide pooled incidence was 161.16 per 100 000 individuals per year (95% CI = 82 to 239). Meta-regressions found a statistically significant higher prevalence of anxiety in patients undergoing primary chemoradiation compared with surgery and increased distress in smokers and advanced tumor staging. European samples exhibited lower prevalence of distress.

**Conclusions:**

Patients with head and neck cancer presented notable prevalence of mental health concerns in all domains. Suicide remains a highly relevant concern. The prevalence of criteria-meeting disorders is significantly lower than clinically relevant symptoms. Investigating the effectiveness of targeted assessments for disorders in highly symptomatic patients is essential.

Tumors of the head and neck region represent a diverse group of cancers including oral cavity, pharynx, larynx, nasal cavity, paranasal sinuses, and salivary gland cancer. These malignancies are a meaningful global health concern across countries of varying levels of development. In 2020, head and neck cancer represented the seventh most common cancer globally, as reported by the Global Cancer Observatory (1). Projections for 2030 suggest an alarming global increase, mainly led by those tumors related to human papillomavirus (2).

Head and neck cancer is associated with particularly relevant levels of emotional distress (3,4). This can be attributed to several factors. This type of cancer is associated with high rates of comorbidity with medical, psychological, and substance abuse disorders (5). Moreover, the impact of the tumor, together with surgical, radiotherapeutic, and systemic treatments, often in combination, can result in a range of functional impairments (6). These encompass difficulties in speech, swallowing, loss of smell and taste, hearing, and even dyspnea (6,7). Additionally, the possibility of physical disfigurement adds a substantial psychological burden (8) and further compromises the quality of life of the affected patients (9).

Indeed, Hammermuller et al. (10) reported high symptomatic scores for depression, anxiety, and fatigue in this population, along with a lower overall quality of life. Wu and colleagues (11) also reported a prevalence of anxiety and depression of more than 25% after a head and neck cancer diagnosis. Suicide is not an exception: Cancer patients are at double the risk of suicide compared with the general population, whereas individuals with head and neck malignancies are 3 times more likely to commit suicide (12).

However, identifying emotional distress in this population remains a considerable challenge. The disease can manifest symptoms such as fatigue, loss of appetite, and sleep disturbances (13,14), effectively masking potential underlying psychiatric conditions. Moreover, clinicians often lack sufficient time for a detailed assessment of this aspect of the illness, coupled with the limited diagnostic skills of many health-care professionals in recognizing such symptoms (15,16); all contribute to the complexity of the situation. To address these challenges, various self-report questionnaires are employed to detect symptoms associated with disorders such as depression (17), anxiety (18), distress (19), insomnia (20), and posttraumatic stress (21). In other studies, structured interviews have been used to diagnose these conditions, following the criteria established in diagnostic manuals (22,23).

Although some prior systematic reviews have explored the prevalence of specific psychological symptoms in cancer patients, such as depression (24) or distress (4), to the best of our knowledge this is the first comprehensive systematic review or meta-analysis that thoroughly investigates all available clinical mental health domains and the factors that influence them in the distinct population of patients with head and neck cancer. Moreover, it is still unclear whether the symptomatic burden observed in this population, as assessed through self-reported scales, reaches the threshold for specific psychiatric disorders.

In this review, we systematically researched the literature to determine meta-analytically the prevalence of psychological symptoms and/or disorders (including depression, anxiety, distress, posttraumatic stress, insomnia, and committed suicide) among patients with head and neck cancer. Additionally, we sought to examine whether this prevalence is influenced by demographic and clinical factors (eg, age, sex, the location of the primary tumor, timing of assessment, type of treatment or comorbidities) or methodological aspects related to diagnosis, including the scale or diagnostic interview, publication year, or publication bias.

## Methods

This study protocol was registered on PROSPERO (registration number: CRD42023441432). The study was conducted in accordance with Preferred Reporting Items for Systematic Reviews and Meta-Analyses (25) (Supplementary Table 1, available online) and Meta-analyses of Observational Studies in Epidemiology checklist (26) (Supplementary Table 2, available online), following EQUATOR Reporting Guidelines (27).

### Search strategy and selection criteria

A systematic literature search was carried out by 2 independent researchers (PJL and CA). Web of Science database (Clarivate Analytics) was searched, incorporating the Web of Science Core Collection, the BIOSIS Citation Index, the KCI Korean Journal Database, MEDLINE, the Russian Science Citation Index, and the SciELO Citation Index as well as Cochrane Central Register of Reviews, and Ovid PsycINFO databases, from inception until August 1, 2023.

The following keywords were used: (“cancer*” OR “neoplasm*” OR “tumour*” OR “tumor*” OR “malignan*”) AND (“head and neck” OR “head & neck” OR “larynx” OR “*pharynx” OR “oral cavity” OR “sinus” OR “cavum” OR “salivary” OR “nasal cavity” OR “tongue” OR “tonsil*”) AND (“suicide*” OR “depress*” OR “anxiety” OR “anxious” OR “insomnia*” OR “post-traumatic” OR “PTSD” OR “stress” OR “distress*”).

Articles identified were first screened as abstracts, and after the exclusion of those that did not meet the inclusion criteria, the full texts of the remaining articles were assessed for eligibility and inclusion. The search was completed by manually searching through references of previously published systematic reviews and meta-analyses on the topic.

Inclusion criteria for the systematic review and meta-analysis were 1) individual studies with original data; 2) focusing on samples containing more than 90% of patients with a histological diagnosis of a head and neck cancer (including oral cavity, oropharynx, hypopharynx, larynx, nasopharynx, paranasal sinuses, nasal cavity, and salivary glands); 3) reporting prevalence about mental health outcomes included in at least 1 of the following categories: anxiety, depression, acute stress or distress, posttraumatic symptoms, sleep disturbances, and suicide; 4) using validated, structured, evaluation scales; 5) nonoverlapping samples (overlap was determined by looking at the inclusion dates and country in which the study was carried out; in case of overlapping, the study with the largest sample was selected); and 6) written in English or Spanish. Exclusion criteria were 1) reviews, clinical cases, study protocols or qualitative studies, conferential proceedings, letters, and commentaries; 2) reporting outcomes on populations with malignancies of different origins than those reported under the inclusion criteria; and 3) including samples already selected based on their psychological distress. Studies including interventions targeting emotional distress were not excluded if a control group with standard treatment was included, for which the data were extracted.

### Data extraction

Two researchers (PJL and CA) independently extracted data from all the included studies. The 2 databases were then cross-checked, and discrepancies were resolved through consensus under the supervision of a senior researcher (AC). A summary of the selected variables included first author and year of publication, country and city, sample size, age (mean [SD]), sex (% female), primary tumor location, cancer stage, treatment intent, type of treatment, mental health domain studied, evaluation tool used, quality assessment (see below), and key findings. When multiple timepoints were available for a particular sample, the measurement immediately following treatment completion (or the closest to it, if the former was not available) was selected.

### Risk of bias (quality) assessment

Risk of bias was assessed using a modified version of the Newcastle–Ottawa Scale for assessing the quality of nonrandomized studies because of the heterogeneity expected in the included studies. Studies were awarded 0-9 points according to their representativeness, exposure, outcomes, comparability, and follow-up period (Supplementary Table 3, available online). Scores of at least 7, 4-6, and less than 4 are considered low, intermediate, and high risk of bias, respectively (28). Interrater agreement between the 2 reviewers was 0.87.

### Strategy for data synthesis

First, we provided a systematic synthesis (Supplementary Table 4, available online) of the findings from the included studies structured around the selected 6 mental health outcomes: depression, anxiety, distress, posttraumatic symptoms, insomnia, and suicide. Second, we performed meta-analyses using, as primary effect size, the prevalence (% and standard error, when available) of mental health outcomes in people with head and neck cancer. For suicide, incidence rate and standard error for each included study was extracted (or calculated where data allowed for it) and then meta-analyzed. As heterogeneity was expected to be high, random-effects meta-analysis models were used for all the studied variables. Meta-regressions using group-level data were performed to determine the effect of age, sex, alcohol and tobacco use, education, employment status, race, civil status, primary tumor location, cancer stage, months since diagnosis, treatment, laryngectomy, pain, and dysphagia, when at least 7 studies provided data. Sensitivity analyses were conducted to estimate the association between the mental health domains and the assessment scale or interview, the continent where the study took place, and the time when the mental health domain was measured (categorized as pretreatment, during treatment, first year after treatment finalization, and more than 1 year after treatment finalization). Heterogeneity among studies was assessed using the *Q* statistic, with the proportion of the total variability in the effect size estimates evaluated using the *I*^2^ index (with an *I*^2^ > 50% representing significant heterogeneity) (29). Publication biases were assessed for the prevalence of symptomatic subjects for each mental health domain by inspecting funnel plots and assessing Egger test (30).

All analyses were conducted using R 4.2.2 (31). The significance level was set at as 2-sided and a *P *value less than .05.

## Results

**Figure 1.**
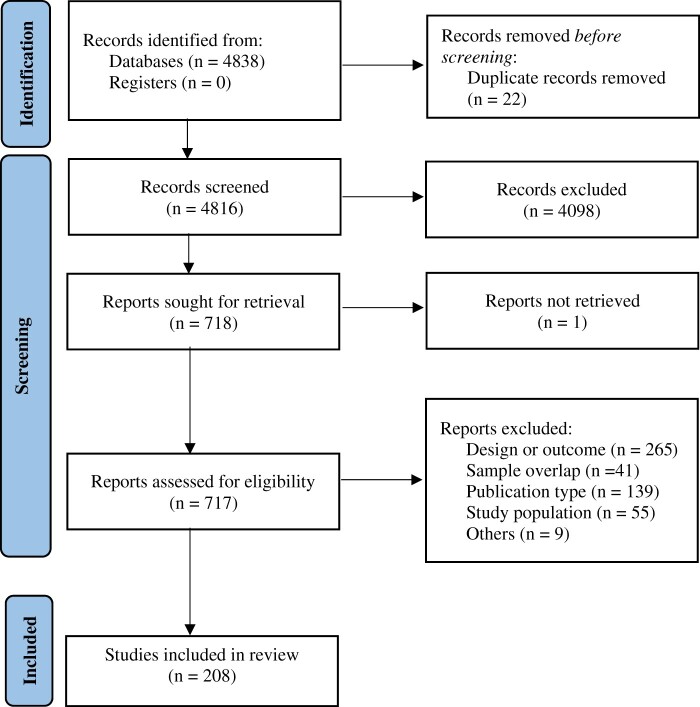
Preferred Reporting Items for Systematic Reviews and Meta-Analysis 2020 flow diagram.

The literature search yielded 4838 citations through electronic database, which were screened for eligibility; 717 articles were assessed in full text, and 509 were excluded. No additional studies were included through manual search. The final database for the systematic review and meta-analysis included 208 studies (Figure 1), encompassing 654 413 patients.

**Figure 2.**
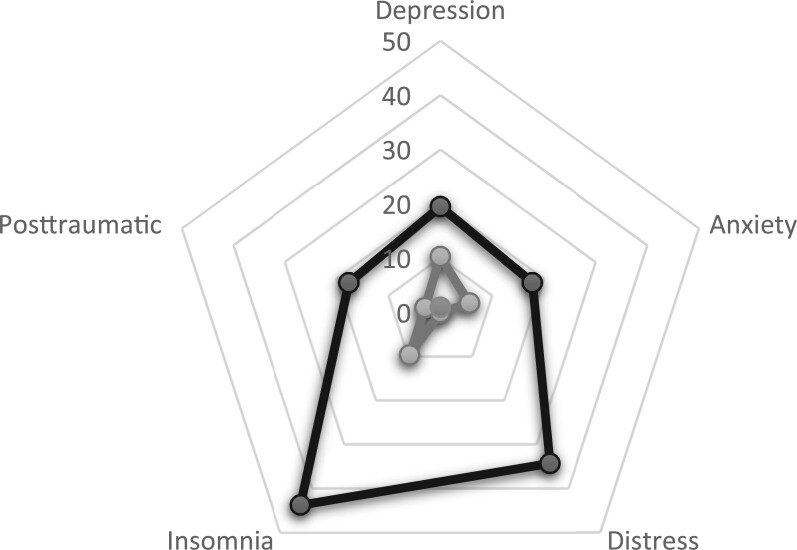
Distribution of the prevalence of disturbances (%) of mental health domains among patients with head and neck cancer, according to clinically significant symptoms (**black**) and disorders (**gray**).

A total of 154 (74.0%) studies focused on depression (127 on depressive symptoms and 27 on depressive disorders), 95 (45.6%) on anxiety (80 on anxiety symptoms and 15 on anxiety disorders), 38 (18.3%) on distress, 9 (4.3%) on insomnia (including 3 on insomnia-related symptoms and 6 on sleep disorders), and 6 (2.9%) on posttraumatic symptoms (3 studies) or disorders (3 studies) (Figure 2). Four additional articles included data on suicide and/or suicidality. The median age of the sample was 60.7 years, and 25.5% were female. Studies included data on patients with a histological diagnosis of primary malignancies of oral cavity (38.7%), oropharynx (27.5%), nasopharynx (7.5%), larynx and hypopharynx (23.4%), or other head and neck sites (2.9%). Of the patients, 36.6% had stage I or II tumors and 63.4% stage III or IV tumors. Of the patients, 21.5% underwent a therapeutical surgery, and 71.5% radiotherapy at some point; 40.3% received primary chemoradiotherapy and 23.2% neoadjuvant or adjuvant chemotherapy. Of the studies, 70% exclusively included patients treated with curative intent. Mean Newcastle–Ottawa scale of the included studies was 6.36 (0.98) (Supplementary Table 4, available online). Heterogeneity was statistically significant for all the studied outcomes (Table 1 and Table 2).

**Table 1.** Prevalence of mental health symptoms across each of the domains and scales studied^a^

Scale	No. studies	Sample size	Prevalence (95% CI)	Heterogeneity	
				*I* ^2^, %	*P*
Depression	127	23 302	0.195 (0.173 to 0.218)	93.3	<.01
HADS-D (32)	68	10 698	0.168 (0.143 to 0.197)	89.4	.01
CES-D (33)	16	2156	0.301 (0.237 to 0.375)	86.5	.01
BDI (34)	14	1853	0.166 (0.123 to 0.222)	81.4	.02
PHQ-9 (35)	11	2639	0.139 (0.110 to 0.174)	81.0	.03
GDS (36)	7	3009	0.380 (0.242 to 0.541)	94.6	<.01
SDS (37)	2	436	0.333 (0.012 to 0.955)	97.4	<.01
BSI-18 (38)	1	125	0.140 (0.089 to 0.213)	NA	NA
QIDS-SR (39)	1	71	0.183 (0.109 to 0.290)	NA	NA
ESAS-D (40)	1	161	0.270 (0.207 to 0.344)	NA	NA
BDI-FS (41)	1	77	0.190 (0.117 to 0.293)	NA	NA
WHO-5 (42)	1	453	0.251 (0.213 to 0.293)	NA	NA
MDI (43)	1	235	0.100 (0.068 to 0.145)	NA	NA
DS (44)	1	1234	0.088 (0.073 to 0.105)	NA	NA
MAAC (45)	1	55	0.403 (0.282 to 0.536)	NA	NA
Mini-5 (46)	1	100	0.490 (0.394 to 0.587)	NA	NA
Anxiety	80	10 478	0.178 (0.145 to 0.215)	90.9	<.01
HADS-A (32)	66	7466	0.162 (0.134 to 0.193)	88.0	<.01
GAD-7 (47)	3	825	0.082 (0.049 to 0.134)	NA	NA
STAI (48)	4	1412	0.511 (0.114 to 0.894)	97.5	<.01
BAI (49)	3	258	0.181 (0.035 to 0.576)	89.2	<.01
SAS (50)	2	315	0.596 (0.000 to 1.000)	98.5	< .01
ESAS-A (40)	1	161	0.230 (0.172 to 0.301)	NA	NA
MAS (51)	1	41	0.412 (0.276 to 0.569)	NA	NA
Distress	38	5057	0.343 (0.298 to 0.390)	88.2	<.01
HADS-T (32)	16	2040	0.306 (0.249 to 0.370)	84.5	<.01
DT (52)	16	2479	0.431 (0.372 to 0.492)	86.1	<.01
ASDS (53)	1	73	0.120 (0.063 to 0.216)	NA	NA
GSI (54)	1	49	0.224 (0.129 to 0.361)	NA	NA
GHQ-12 (55)	1	28	0.570 (0.386 to 0.737)	NA	NA
GHQ-20 (56)	1	204	0.314 (0.254 to 0.381)	NA	NA
GHQ-30 (57)	1	135	0.150 (0.099 to 0.221)	NA	NA
Hornheide Questionnaire (58)	1	49	0.188 (0.102 to 0.322)	NA	NA
Insomnia	3	667	0.438 (0.358 to 0.522)	0.00	.63
PSQI (59)	3	667	0.438 (0.358 to 0.522)	0.00	.63
Posttraumatic stress disorder	3	180	0.177 (0.061 to 0.413)	61.5	.07
PCL-C (60)	1	93	0.118 (0.067 to 0.201)	NA	NA
PSS-SR (61)	1	65	0.190 (0.112 to 0.304)	NA	NA
IES-R (62)	1	22	0.320 (0.161 to 0.535)	NA	NA

a ASDS = Acute Stress Disorder Scale; BAI = Beck Anxiety Inventory; BDI = Beck Depression Inventory; BDI-FS = Beck Depression Inventory–Fast Screen; BSI-18 = Brief Symptom Inventory–18; CES-D = Center for Epidemiological Studies Depression Scale; CI = confidence interval; DS = Depressive Scale; DT = Distress Thermometer; ESAS = Edmonton Symptom Assessment Symptom; GAD-7 = General Anxiety Disorder–7; GDS = Geriatric Depression Scale; GHQ = General Health Questionnaire; GSI = Overall Psychological Distress; HADS = Hospital Anxiety and Depression Scale; IES-R = Impact of Events Scale–Revised; MAAC = Mental Adjustment to Cancer; MAS = Manifest Anxiety Scale; MDI = Major Depression Inventory; Mini-5 = Mini-International Neuropsychiatric Interview; NA = not applicable; PCL-C = Posttraumatic Stress Disorder Checklist–Civilian Version; PHQ = Patient Health Questionnaire; PSQI = Pittsburgh Sleep Questionnaire Inventory; PSS-SR = Posttraumatic Stress Disorder Scale-Self Report; QIDS-SR = Quick Inventory of Depressive Symptoms-Self-Assessment; SAS = Zung Self-Rating Anxiety Scale; SDS Zung = Self-Rating Depression Scale; STAI = State Trait Anxiety Inventory; WHO-5 = World Health Organization–Five Well-Being Index.

**Table 2.** Prevalence of mental disorders across each of the domains and scales studied^a^

Diagnostic criteria	No. studies	Sample size	Prevalence (95% CI)	Heterogeneity	
				*I* ^2^, %	*P*
Depression	27	331 653	0.103 (0.079 to 0.133)	99.5	<.01
* DSM-IV* (63)	14	1886	0.107 (0.068 to 0.163)	80.0	<.01
* ICD-9* (64)	11	328 936	0.091 (0.068 to 0.163)	99.8	<.01
* ICD-10* (65)	1	771	0.149 (0.126 to 0.176)	NA	NA
SADS (66)	1	60	0.200 (0.117 to 0.320)	NA	NA
Anxiety	15	215 368	0.056 (0.029 to 0.105)	99.5	<.01
* DSM-IV* (63)	8	1176	0.061 (0.028 to 0.131)	84.7	<.01
* ICD-9* (64)	6	213 421	0.048 (0.011 to 0.184)	99.8	<.01
* ICD-10* (65)	1	771	0.120 (0.098 to 0.144)	NA	NA
Insomnia	6	67 364	0.096 (0.016 to 0.406)	99.4	<.01
* DSM-IV* (63)	5	433	0.153 (0.028 to 0.537)	96.9	<.01
* ICD-9* (64)	1	66 931	0.009 (0.009 to 0.010)	NA	NA
Posttraumatic stress disorder	3	344	0.010 (0.000 to 0.848)	66.5	.05
* DSM-IV* (63)	3	344	0.010 (0.000 to 0.848)	66.5	.05

a CI = confidence interval; *DSM-IV* = *Diagnostic and Statistical Manual of Mental Disorders–4th Edition*; *ICD* = *International Classification of Diseases*; NA = not applicable; SADS = Schedule for Affective Disorders and Schizophrenia.

### Depression

Depressive symptoms prevalence was reported in 127 studies, including a total sample of 23 302 participants. Multiple evaluation scales were used, including Hospital Anxiety and Depression Scale (HADS) Depression Subscale (k = 68) (32), Centre for Epidemiologic Studies Depression Scale (k = 16) (33), Beck Depression Inventory (k = 14) (34), Patient Health Questionnaire–9 (k = 11) (35), and Zung Self-Rating Depression Scale (k = 2) (37), among others. The pooled prevalence of clinically significant depressive symptoms was 0.195 (95% confidence interval [CI] = 0.173 to 0.218). Prevalence varied widely depending on the scale used, from 0.490 with Mini-International Neuropsychiatric Interview (67) to 0.088 with Depressive Scale (44). The funnel plot and Egger test suggested the presence of a publication bias (*t* = −3.83, *P* < .01). After performing trim and fill method corrections, depressive symptoms prevalence meaningfully increased (corrected prevalence = 0.282, 95% CI = 0.249 to 0.317) (Supplementary Figure 1, A, available online).

Depressive disorders were assessed using diagnostic interviews, including the *Diagnostic and Statistical Manual of Mental Disorders, Fourth Edition* (*DSM-IV*; k = 14) (63), the *International Classification of Diseases*, *Ninth Revision* (*ICD-9*; k = 11) and *Tenth Revision* (*ICD-10*; k = 1) (65,68), and the Schedule for Affective Disorder and Schizophrenia (k = 1) (66), in 27 articles encompassing 331 653 patients. The pooled prevalence of depression disorder was 0.103 (95% CI = 0.079 to 0.133) (Supplementary Figure 2, A, available online). Again, prevalence varied depending on the scale used, from 0.596 with Schedule for Affective Disorder and Schizophrenia to 0.091 with *ICD-9*. No evidence of publication bias was found (Supplementary Figure 1, B, available online).

Subgroup and meta-regression analyses revealed no statistically significant differences for any of the studied variables or for depressive symptoms or for depressive disorders (Supplementary Tables 6 and 7, available online respectively).

### Anxiety

Anxiety symptom prevalence was examined in 80 studies, involving a total of 10 478 participants. Various assessment scales were employed, such as the HADS Anxiety Subscale (k = 66) (32), the Generalized Anxiety Disorder Assessment–7; k = 3) (47), the State-Trait Anxiety Inventory (k = 4) (48), and the Zung Self-Rating Anxiety Scale (k = 2) (50), among others. The pooled prevalence of clinically significant anxiety symptoms was 0.178 (95% CI = 0.145 to 0.215), ranging from 0.596 when using the Zung Self-Rating Anxiety Scale to 0.082 with the Generalized Anxiety Disorder Assessment–7. Meta-regressions found statistically significant lower prevalence of anxiety symptoms in samples where more people underwent surgical procedures (β = −0.685, 95% CI = −1.367 to −0.003) and laryngectomy (β = −1.279, 95% CI = −2.205 to −0.353), whereas higher prevalence of symptoms were found in samples where more people underwent primary chemoradiation (β = 1.138, 95% CI = 0.169 to 2.107) (Supplementary Table 6, available online). No evidence of publication bias was found (Supplementary Figure 1, C, available online).

Anxiety disorders were assessed using *DSM-IV* (k = 8), *ICD-9* (k = 6), and *ICD-10* (k = 1) in 15 studies, including a total of 215 368 patients with head and neck cancer. Their pooled prevalence was 0.056 (95% CI = 0.029 to 0.105) (Supplementary Figure 2, B, available online). Prevalence of these disorders presented statistically significant variations according to when the assessment was performed, with higher prevalence of anxiety disorders before the onset of the oncological treatment (pooled prevalence 0.103, 95% CI = 0.054 to 0.190) and more than 1 year after the treatment had finished (pooled prevalence 0.178, 95% CI = 0.011 to 0.812) (Supplementary Table 7, available online). Meta-regressions revealed no statistically significant differences for any of the studied variables. No evidence of publication bias was found (Supplementary Figure 1, D, available online).

### Distress

**Figure 3.**
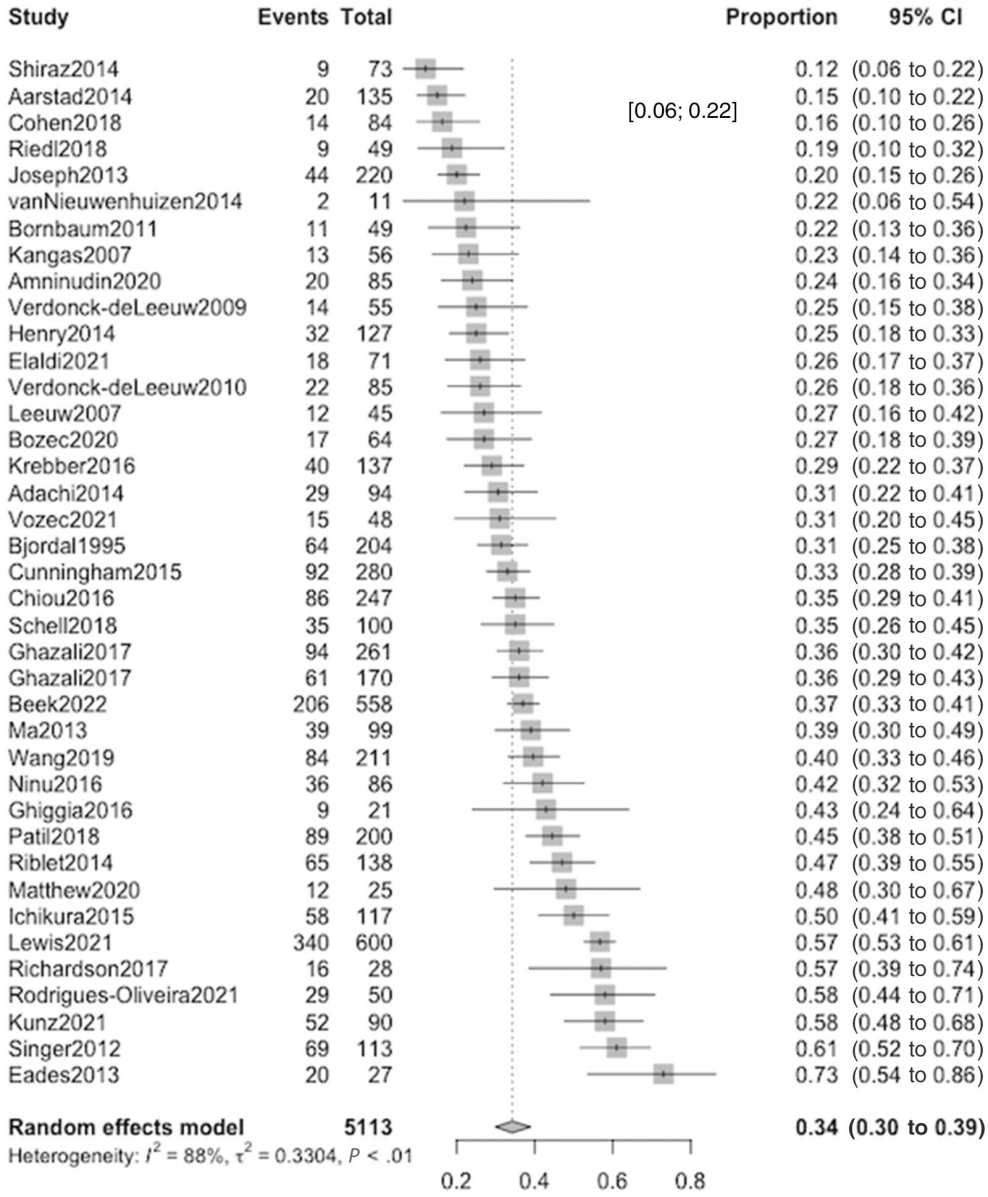
Forest plot for clinically significant distress. CI = confidence interval.

Prevalence of distress symptoms was reported in 38 studies, encompassing a total of 5057 participants. Multiple assessment scales were used, including the HADS total score (k = 16) (32), the Distress Thermometer (k = 16) (52), the Adapted Symptom Distress Scale (k = 1) (53), and different versions of the General Health Questionnaire (k = 1) (55). The combined prevalence of clinically significant distress symptoms was 0.343 (95% CI = 0.298 to 0.390), ranging from 0.570 with General Health Questionnaire–12 to 0.120 with Adapted Symptom Distress Scale (Figure 3; Supplementary Figure 2, H, available online). Meta-regressions found statistically significant higher prevalence of distress among samples with higher rates of tobacco use (β = 1.659, 95% CI = 0.290 to 3.027) and advanced stages (β = 1.283, 95% CI = 0.177 to 2.389). Samples with more females (β = −2.519, 95% CI = −4.170 to −0.869), more patients with higher levels of education (β = −3.703, 95% CI = −6.894 to −0.512), and earlier stages of the disease (β = −1.823, 95% CI = −2.884 to −0.762) presented lower prevalence of distress (Supplementary Table 6, available online). Prevalence of distress presented statistically significant variations according to the continent of the sample (with European samples presenting the lowest prevalence of distress at 0.304 [95% CI = 0.254 to 0.359]) and the timing of the assessment (with distress decreasing as time from diagnosis and treatment passed) (Supplementary Table 7, available online). No evidence of publication bias was found (Supplementary Figure 1, E, available online).

### Insomnia

Insomnia-related relevant symptoms were assessed in 3 studies (n = 667 patients) using the Pittsburgh Sleep Quality Index (59), with a pooled prevalence of 0.438 (95% CI = 0.358 to 0.522). Insomnia disorder was also assessed in 6 studies (n = 67 364 patients) using *DSM-IV* (k = 5) and *ICD-9* (k = 1), with an overall prevalence of 0.096 (95% CI = 0.016 to 0.406) (Supplementary Figures 2, C and D, available online). Not enough data were available to perform any meta-regression or subgroup analysis.

### Posttraumatic symptoms and disorder

Posttraumatic symptoms were studied in 3 studies, with a total sample size of 180 patients and a pooled prevalence of 0.177 (95% CI = 0.061 to 0.413), while posttraumatic stress disorder was assessed in 3 studies (n = 344) using *DSM-IV*, with an overall prevalence of 0.010 (95% CI = 0.000 to 0.848) (Supplementary Figure 2, E and F, available online). Not enough data were available to perform any meta-regression or subgroup analysis.

### Suicide

Suicide incidence among patients with head and neck cancer was reported in 4 studies, including a total of 1 976 569 person-years (Supplementary Table 5, available online). Overall pooled incidence of suicide was 161.16 suicides per 100 000 individuals per year (95% CI = 82.88 to 239.43) (Supplementary Figure 2, G, available online). Not enough data were available to meta-analyze the standardized mortality risk or any other relative risk outcome.

## Discussion

This is the first systematic review and meta-analysis to date to comprehensively assess the prevalence of mental health symptoms and disorders among patients with head and neck cancer.

In our study, 19.5% of patients with head and neck cancer were found to report clinically significant depressive symptoms through self-reported scales, a percentage that decreased to 10.3% when considering diagnosed depressive disorders through clinical interviews. These findings closely align with those reported by Krebber et al. (24) in a meta-analysis that encompassed samples of cancer patients with various primary tumor types and far surpass the prevalence of major depression disorders observed in the general population, which hover around 4% (69). Surprisingly, in our meta-analysis the prevalence of depressive symptoms or disorders does not appear to be influenced by variables such as tumor location, tumor stage, treatment type, or even well-known depression-inducing factors like tobacco (70) or alcohol (71) consumption. There are several possible explanations for these findings. Depression is a complex mental disorder that can be influenced by individual factors and external circumstances (72), with many of them being idiosyncratic to the individual: genetic factors (73), coping styles and distorted thinking patterns (74), traumatic life events (75), or lack of support systems (76), among many others. Moreover, depression takes a notable time to develop [and to be considered as a disorder, according to *DSM* (77) and *ICD* (68)], which could help explain why depressive symptoms could be less prone to be triggered by external factors.

Anxiety symptoms appeared to be clinically significant in 17.8% of the patients with head and neck cancer, whereas 5.6% met criteria for an anxiety disorder. The prevalence of anxious symptoms was positively correlated with primary chemoradiation and negatively correlated surgical procedures and laryngectomy. Although surgical procedures in general (78) and laryngectomy in particular (79) have been described as highly distressing procedures, our results could be indirectly reflecting the underlying overall prognosis of the sample (80). Surgical approaches, especially highly invasive procedures like laryngectomy, are typically reserved for patients who are generally fitter and have a better prognosis. Conversely, patients treated with primary chemoradiation tend to have more advanced tumor stages (which contraindicate surgery) and poorer overall health (81-83). Therefore, we believe that the results of our study may reflect that higher levels of anxiety are associated with an unfavorable prognosis. The results of our meta-analysis regarding distress appear to support this hypothesis. The pooled prevalence of distress in the sample is 34.3%, with higher stress levels in advanced tumor stages and lower levels as the measurement moves further from diagnosis and treatment. Interestingly, European samples exhibited a significantly lower distress prevalence compared with those from other continents. Although this may be influenced by various factors, this phenomenon may be related to financial toxicity, defined as the direct and indirect costs associated with cancer diagnosis, treatment, and care (84). In Europe, predominantly publicly funded health-care systems often shield patients from catastrophic medical expenses while also providing effective treatments and overall care (85).

As for insomnia, the prevalence of symptoms was 43.8%, with 9.6% of the sample meeting criteria for a disorder. Sleep disturbances are a common complaint among patients with head and neck cancer (86), often associated with pain (87), xerostomia (88), and radiation therapy–related obstructive sleep apnea (89). Insomnia is closely linked to quality of life (90), anxiety (91), depression (92), and alcohol consumption (93). Many of its causal factors are potentially modifiable, making it a promising target for improving the mental health of patients with head and neck cancer. Regarding posttraumatic symptoms, 17.7% exhibited clinically significant symptoms, although the pooled prevalence of posttraumatic stress disorder was approximately 1%. Unfortunately, the wide confidence intervals in the latter outcome do not allow for reliable conclusions.

Finally, the overall incidence of suicide in the analyzed sample was 161.16 suicides per 100 000 individuals per year. Suicide is a complex phenomenon influenced by individual, cultural, religious, economic, and health-care access factors (94). Therefore, explaining its fluctuations through a single event such as cancer is challenging. Furthermore, 2 of the samples included in this meta-analysis come from specific populations with a high risk of bias because of the relationship with high rates of suicide: war veterans (95) and the elderly population (96). However, and although we did not obtain sufficient data to analyze standardized mortality ratios or relative risks, all data point to the incidence of suicide in patients with head and neck cancer being higher than that in the general population (12) and other cancer patients (97).

The conclusions drawn from these findings are manifold. It is evident that head and neck cancers meaningfully impact the mental health of those affected, extending across multiple domains, as our findings point out. However, it is noteworthy that the prevalence of clinically relevant symptoms is much higher compared with the prevalence of criterion-meeting mental disorders for all the studied domains. Self-reported questionnaires could provide an overestimation of the actual percentage of patients with a psychiatric disorder among this population, as previous studies on this field corroborate (24). Indeed, most measures of depression or anxiety severity are based on the number of reported symptoms, with threshold scores often used to classify individuals as healthy or ill. This would be valid if depression, or anxiety, were single conditions and all their symptoms equally good severity indicators. However, specific symptoms like concentration problems, insomnia, or anhedonia are distinct phenomena differing from each other in dimensions such as their biological mechanisms or risk factors (98). Reporting specific symptom profiles among cancer patients, instead of disorders as dichotomic variables, could provide valuable information on their causes, their relationship with the underlying oncological process, and best ways to address them.

Systematic assessment of mental health should be an integral part of oncological care in patients with head and neck cancer, much like the assessment of pain or other physical discomfort, for which we suggest a stepped-care approach. Self-reported questionnaires can be an effective initial screening tool to identify patients with clinically significant symptoms (99). Systematic screening for emotional distress in patients with some cancer types, in addition to present evidence in the early detection of mental disorders (100), promotes equal access to psychological services, in contrast with a referral system solely reliant on the initiative of doctors or patients (101).

For those patients who exhibit such distress, the evaluation should be completed with a detailed interview to determine if they indeed present a mental disorder in which case they should be referred to the appropriate specialist for the necessary psychotherapeutic and/or pharmacological treatment at the earliest stages. As for patients experiencing evident symptoms but who do not meet criteria for a disorder, close monitoring of symptoms is recommended. Additionally, group psychotherapeutic approaches have shown strong evidence in alleviating distress in oncologic patient samples while maintaining cost-effectiveness (102,103).

Notwithstanding all the above, future research should aim to study this subgroup of patients with subclinical mental health impairment to characterize their prognosis and trajectories, along with the effectivity of preventive and therapeutic interventions.

This study has several important strengths. To the best of the authors’ knowledge, it represents the most extensive meta-analysis to date concerning mental health outcomes in patients with head and neck cancer. Moreover, it explores facets of mental health that have received less attention in prior literature, such as insomnia or suicide. Additionally, it categorizes the evidence into 2 important categories—symptoms and disorders—thus facilitating valuable comparisons between the 2 concepts. The inclusion of samples from more than 29 countries spanning 6 continents ensures high generalizability.

The findings of this study must be interpreted in light of certain limitations; the primary limitation is the substantial heterogeneity among the assessed outcomes. Various scales have been employed to evaluate each domain, assessing slightly different aspects of each mental health outcome and using different cutoff points. The populations within each study exhibit varying frequencies, reflecting the heterogeneity observed in head and neck cancers, as well as different stages of the disease. Most of the included studies do not provide stratified data for each primary location or type of treatment, which is why the effect of these variables could only be analyzed through meta-regressions, which could incur into ecological bias (104). There was not enough data about psychiatric antecedents and drug use to study their influence either. Furthermore, the cross-sectional nature of the included measurements limits our understanding of the evolution of mental health outcomes throughout the oncological process. In studies that analyze long-term survivors, we cannot rule out the presence of survival bias. Future studies should longitudinally assess the evolution of emotional distress to identify the most intervention-sensitive points.

Our findings reveal that patients with head and neck cancer are particularly vulnerable to a spectrum of mental health symptoms and disorders, with great proportions of them experiencing depressive symptoms, anxiety, distress, insomnia, posttraumatic symptoms, and even suicide. Self-reported questionnaires, however, could provide an overestimation of the prevalence of disorders among this population. Future research should focus on longitudinal evaluations to identify intervention-sensitive points and develop targeted interventions that enhance the mental health and quality of life of individuals facing head and neck cancer.

## Supplementary Material

pkae031_Supplementary_Data

## Data Availability

The data that support the findings of this study are available from the corresponding author, PJL, on reasonable request.
