# Supplementary material for: A comprehensive examination of mental health in patients with head and neck cancer: systematic review and meta-analysis
Source: JNCI Cancer Spectr. 2024 May 3;8(3):pkae031. doi: 10.1093/jncics/pkae031 (PMC11149920; doi:10.1093/jncics/pkae031)
Supplement: pkae031_Supplementary_Data [file pkae031_supplementary_data.zip › suppl_data/Supplementary Material_Final -Track Changes.docx]

**SUPPLEMENTARY MATERIAL**

**Supplementary Table 1:** PRISMA statement and checklist…………….……...................page 2-4

**Supplementary Table 2**: MOOSE Statement checklist…………………………..……….page 5-6

**Supplementary Table 3**: Quality assessment: Newcastle-Ottawa Scale (NOS) for Cohort Studies…………………………………………………………………………………..….....page 7

**Supplementary Table 4**: Main characteristics of the included studies……...….…….….page 8-17

**Supplementary Table 5**: Suicide incidence of the included studies………….…………….page 18

**Supplementary Figure 1**: Funnel plots for publication bias…………………………….page 19-20

**Supplementary Table 6**: Meta-regressions for the studied outcomes………….……….page 21-23

**Supplementary Table 7**: Subgroup analyses for the studied outcomes...……………….page 24-25

**Supplementary Figure 2**: Forest plots for the studied outcomes……….……………….page 26-28

**Supplementary References**……….……………………………..……...……………….page 29-53

**Supplementary Table 1: Prisma statement and checklist**

| **Section/Topic** | **Item #** | **Checklist item** | **Page** |
| --- | --- | --- | --- |
| **TITLE** | | | |
| Title | 1 | Identify the report as a systematic review. | 1 |
| **ABSTRACT** |  |  |  |
| Abstract | 2 | Provide a structured summary including, as applicable: background; objectives; data sources; study eligibility criteria, participants, and interventions; study appraisal and synthesis methods; results; limitations; conclusions and implications of key findings; systematic review registration number. | 2 |
| **INTRODUCTION** | | | |
| Rationale | 3 | Describe the rationale for the review in the context of existing knowledge. | 3 |
| Objectives | 4 | Provide an explicit statement of the objective(s) or question(s) the review addresses. | 4 |
| **METHODS** | | | |
| Eligibility criteria | 5 | Specify the inclusion and exclusion criteria for the review and how studies were grouped for the syntheses. | 5 |
| Information sources | 6 | Specify all databases, registers, websites, organisations, reference lists and other sources searched or consulted to identify studies. Specify the date when each source was last searched or consulted. | 4,5 |
| Search strategy | 7 | Present the full search strategies for all databases, registers and websites, including any filters and limits used. | 4,5 |
| Selection process | 8 | Specify the methods used to decide whether a study met the inclusion criteria of the review, including how many reviewers screened each record and each report retrieved, whether they worked independently, and if applicable, details of automation tools used in the process. | 5 |
| Data collection process | 9 | Specify the methods used to collect data from reports, including how many reviewers collected data from each report, whether they worked independently, any processes for obtaining or confirming data from study investigators, and if applicable, details of automation tools used in the process. | 5,6 |
| Data items | 10 | List and define all outcomes for which data were sought and if any assumptions were made about any missing or unclear information. | 5,6 |
| Study risk of bias assessment | 11 | Specify the methods used to assess risk of bias in the included studies, including details of the tool(s) used, how many reviewers assessed each study and whether they worked independently, and if applicable, details of automation tools used in the process. | 5-7 |
| Effect measures | 12 | Specify for each outcome the effect measure(s) (e.g. risk ratio, mean difference) used in the synthesis or presentation of results. | 6,7 |
| Synthesis methods | 13 | Describe the processes used to decide which studies were eligible for each synthesis. Describe any methods required to prepare the data for presentation or synthesis, such as handling of missing summary statistics, or data conversions. Describe any methods used to tabulate or visually display results of individual studies and syntheses. Describe any methods used to synthesize results and provide a rationale for the choice(s). Describe the model(s), method(s) to identify the presence and extent of statistical heterogeneity, and software package(s) used, any methods used to explore possible causes of heterogeneity among study results (e.g. subgroup analysis, meta-regression) and any sensitivity analyses conducted to assess robustness of the synthesized results. | 5-7 |
| Reporting bias assessment | 14 | Describe any methods used to assess risk of bias due to missing results in a synthesis (arising from reporting biases). | 5 |
| Certainty assessment | 15 | Describe any methods used to assess certainty (or confidence) in the body of evidence for an outcome. | 5-7 |
| **RESULTS** | | | |
| Study selection | 16 | Describe the results of the search and selection process, from the number of records identified in the search to the number of studies included in the review, ideally using a flow diagram. Cite studies that might appear to meet the inclusion criteria, but which were excluded, and explain why they were excluded. | 7-11, 20 |
| Study characteristics | 17 | Cite each included study and present its characteristics. | 7-11, 17-19 |
| Risk of bias in studies | 18 | Present assessments of risk of bias for each included study. | Supp 8-20 |
| Results of individual studies | 19 | For all outcomes, present, for each study: (a) summary statistics for each group (where appropriate) and (b) an effect estimate and its precision (e.g. confidence/credible interval), ideally using structured tables or plots. | 17-19 |
| Results of syntheses | 20 | For each synthesis, briefly summarise the characteristics and risk of bias among contributing studies. Present results of all statistical syntheses conducted. If meta-analysis was done, present for each the summary estimate and its precision (e.g. confidence/credible interval) and measures of statistical heterogeneity. If comparing groups, describe the direction of the effect. Present results of all investigations of possible causes of heterogeneity among study results and all sensitivity analyses conducted to assess the robustness of the synthesized results. | 7, Supp 8-25 |
| Reporting biases | 21 | Present assessments of risk of bias due to missing results (arising from reporting biases) for each synthesis assessed. | 7, Supp 8-20 |
| Certainty of evidence | 22 | Present assessments of certainty (or confidence) in the body of evidence for each outcome assessed. | 17-19 |
| **DISCUSSION** | | | |
| Discussion | 23 | Provide a general interpretation of the results in the context of other evidence. Discuss any limitations of the evidence included in the review, any limitations of the review processes used and the implications of the results for practice, policy, and future research. | 11-15 |
| **OTHER INFORMATION** | | | |
| Registration and protocol | 24 | Provide registration information for the review, including register name and registration number, or state that the review was not registered. Indicate where the review protocol can be accessed, or state that a protocol was not prepared. Describe and explain any amendments to information provided at registration or in the protocol. | 4 |
| Support | 25 | Describe sources of financial or non-financial support for the review, and the role of the funders or sponsors in the review. | 16 |
| Competing interests | 26 | Declare any competing interests of review authors. | 16 |
| Availability of data, code and other materials | 27 | Report which of the following are publicly available and where they can be found: template data collection forms; data extracted from included studies; data used for all analyses; analytic code; any other materials used in the review. | 16 |

For more information, visit: <http://www.prisma-statement.org/>

**Supplementary Table 2: MOOSE Statement - Reporting Checklist for Authors, Editors, and Reviewers of Meta-analyses of Observational Studies**

| **Reporting Criteria** | **Reported (Yes/No)** | **Reported on Page** |
| --- | --- | --- |
| **Reporting of Background** |  |  |
| Problem definition | Yes | 2-4 |
| Hypothesis statement | Yes | 3,4 |
| Description of Study Outcome(s) | Yes | 4,6 |
| Type of exposure or intervention used | Yes | 5 |
| Type of study design used | Yes | 2-6 |
| Study population | Yes | 5 |
| **Reporting of Search Strategy** |  |  |
| Qualifications of searchers (eg, librarians and investigators) | Yes | 1,4 |
| Search strategy, including time period included in the synthesis and keywords | Yes | 4,5 |
| Effort to include all available studies, including contact with authors | Yes | 4,5 |
| Databases and registries searched | Yes | 4,5 |
| Search software used, name and version, including special features used (eg, explosion) | Yes | 4,7 |
| Use of hand searching (eg, reference lists of obtained articles) | Yes | 4-6 |
| List of citations located and those excluded, including justification | Yes | 4,5, 20 |
| Method for addressing articles published in languages other than English | Yes | 5 |
| Method of handling abstracts and unpublished studies | Yes | 4-7 |
| Description of any contact with authors | N.a. | 4-6, 20 |
| **Reporting of Methods** |  |  |
| Description of relevance or appropriateness of studies assembled for assessing the hypothesis to be tested | Yes | 4-6 |
| Rationale for the selection and coding of data (eg, sound clinical principles or convenience) | Yes | 4-7 |
| Documentation of how data were classified and coded (eg, multiple raters, blinding, and interrater reliability) | Yes | 4-7 |
| Assessment of confounding (eg, comparability of cases and controls in studies where appropriate | Yes | N/A |
| Assessment of study quality, including blinding of quality assessors; stratification or regression on possible predictors of study results YES 5 | Yes | 5, Supp 8-20 |
| Assessment of heterogeneity | Yes | 6,7, 17-19, Supp 20-25 |
| Description of statistical methods (eg, complete description of fixed or random effects models, justification of whether  the chosen models account for predictors of study results, dose-response models, or cumulative meta-analysis) in sufficient detail to be replicated | Yes | 6,7, 17-19, Supp 21-28 |
| Provision of appropriate tables and graphics | Yes | 17-21, Supp 8-28 |
| **Reporting of Results** |  |  |
| Table giving descriptive information for each study included | Yes | 17-19 |
| Results of sensitivity testing (eg, subgroup analysis) | Yes | 7-11, 17-19 |
| Indication of statistical uncertainty of findings | Yes | 7-11, 17-19 |
| **Reporting of Discussion** |  |  |
| Quantitative assessment of bias (eg, publication bias) | Yes | 7-11, Supp 8-20 |
| Justification for exclusion (eg, exclusion of non–English-language citations) | Yes | 20 |
| Assessment of quality of included studies | Yes | 7, Supp 8-20 |
| **Reporting of Conclusions** |  |  |
| Consideration of alternative explanations for observed results | Yes | 11-15 |
| Generalization of the conclusions (ie, appropriate for the data presented and within the domain of the literature review) | Yes | 14,15 |
| Guidelines for future research | Yes | 14,15 |
| Disclosure of funding source | Yes | 16 |

**Supplementary Table 3: Quality assessment: Newcastle-Ottawa Scale (NOS) for Cohort Studies**

Quality of the included studies was assessed using the Newcastle-Ottawa Scale (NOS) for Cohort Studies due to the heterogeneity expected in the included studies. The following assessment scores were used:

| **Criteria** | **Maximum Score** |
| --- | --- |
| Representativeness of exposed cohort | ★ |
| Selection of the non-exposed cohort | ★ |
| Ascertainment of exposure | ★ |
| Demonstration that outcome of interest was not present at start of study | ★ |
| Comparability of cohorts on the basis of the design or analysis controlled for confounders | ★ ★ |
| Assessment of outcome | ★ |
| Was follow-up long enough for outcomes to occur | ★ |
| Adequacy of follow-up of cohorts | ★ |

**Supplementary Table 4: Main characteristics of the included studies**

| **Study** | **City (Country)** | **Location** | **N** | **Age:**  **mean** **±SD** | **% Female** | **NOS** | **Scale (Domain)** |
| --- | --- | --- | --- | --- | --- | --- | --- |
| Bastos et al., 2018 ^1^ | Sao Paulo (Brazil) | Multitumour | 93 | 56.7 ±10.9 | 0.12 | 4/9 | BAI (Anxiety) |
| Menezes et al., 2021 ^2^ | Minas Gerais (Brazil) | Multitumour | 55 | 61.5 ±10.8 | 0.21 | 6/9 | BAI (Anxiety), HADS-A (Anxiety), HADS-D (Depression) |
| Birkhaug et al., 2002 ^3^ | Multiple (Norway) | Larynx & Hypopharynx | 104 | 66 ±10 | 0.14 | 8/9 | BDI (Depression) |
| Byrne et al., 1993 ^4^ | Dublin (Ireland) | Larynx ± Hypopharynx | 20 | N/A | 0.1 | 4/9 | BDI (Depression) |
| Chan et al., 2011 ^5^ | Washington (USA) | Multitumour | 77 | N/A | 0.23 | 6/9 | BDI (Depression) |
| Chang et al., 2012 ^6^ | Seoul (South Korea) | Multitumour | 56 | 61 | 0.23 | 6/9 | BDI (Depression) |
| Chawla et al., 1999 ^7^ | New Delhi (India) | Multitumour | 50 | 51.42 ±7.68 | 0 | 6/9 | BDI (Depression) |
| Chiou et al., 2013 ^8^ | Chiayi (Taiwan) | Multitumour | 73 | 50 ±9 | 0.1 | 7/9 | BDI (Depression) |
| Dantonio et al., 1998 ^9^ | Scottsdale (USA) | Multitumour | 50 | 62 | 0.24 | 6/9 | BDI (Depression) |
| El-Deiry et al., 2005 ^10^ | Iowa (USA) | Multitumour | 54 | 55.9 | 0.31 | 7/9 | BDI (Depression) |
| Givens et al., 2009 ^11^ | Iowa (USA) | Multitumour | 104 | 55.7 | 0.269 | 7/9 | BDI (Depression) |
| Haisfield-Wolfe et al., 2011 ^12^ | Baltimore (USA) | Larynx & Hypopharynx | 21 | 59.2 ±9.2 | 0.14 | 6/9 | BDI (Depression) |
| Howren et al., 2010 ^13^ | Iowa (USA) | Multitumour | 355 | 60 ±12.5 | 0.373 | 7/9 | BDI (Depression) |
| Howren et al., 2012 ^14^ | Iowa (USA) | Multitumour | 249 | 60.7 ±12.3 | 0.357 | 8/9 | BDI (Depression) |
| Kim et al., 2015 ^15^ | Seoul (South Korea) | Multitumour | 241 | 61 | 0.15 | 7/9 | BDI (Depression) |
| Pytel et al., 2023 ^16^ | Wroclaw (Poland) | Multitumour | 85 | N/A | 0.353 | 4/9 | BDI (Depression) |
| Terrell et al., 1998 ^17^ | Multiple (USA) | Larynx & Hypopharynx | 46 | 58.3 | 0.087 | 8/9 | BDI (Depression) |
| Sarafim-Silva et al., 2018 ^18^ | Sao Paulo (Brazil) | Multitumour | 110 | N/A | 0.118 | 7/9 | BDI (Depression), BAI (Anxiety) |
| Karnell et al., 2006 ^19^ | Iowa (USA) | Multitumour | 235 | N/A | 0.318 | 7/9 | BDI (Depression), HADS-A (Anxiety), HADS-D (Depression) |
| Bakhshaie et al., 2019 ^20^ | Houston (USA) | Multitumour | 125 | 58.6 ±10.43 | 0.1342 | 6/9 | BSI-HADS-D (Depression)8 (Depression) |
| Astrup et al., 2015 ^21^ | Oslo (Norway) | Multitumour | 133 | 60 ±11 | 0.29 | 6/9 | CES-D (Depression) |
| Van Der Schroeff et al., 2007 ^22^ | Utrecht (Netherlands) | Multitumour | 57 | N/A | 0.23 | 6/9 | CES-D (Depression) |
| Nightingale et al., 2014 ^23^ | Gainesville (USA) | Oropharynx | 10 | 60 ±9.14 | 0.2 | 5/9 | CES-D (Depression) |
| Chhabria et al., 2017 ^24^ | Florida (USA) | Oropharynx | 81 | 58.2 | 0.233 | 7/9 | CES-D (Depression) |
| Irish et al., 2009 ^25^ | Toronto (Canada) | Oral cavity | 42 | 60.7 ±153 | 0.29 | 7/9 | CES-D (Depression) |
| Katz et al., 2002 ^26^ | Toronto (Canada) | Multitumour | 82 | 58.8 | 0.305 | 7/9 | CES-D (Depression) |
| Leeuw et al., 2000-A ^27^ | Utrecht (Netherlands) | Multitumour | 197 | 58.8 ±10.7 | 0.22 | 5/9 | CES-D (Depression) |
| Monga et al., 1997 ^28^ | Houston (USA) | Multitumour | 55 | 65 | 0.02 | 6/9 | CES-D (Depression) |
| Morse et al., 2010 ^29^ | Multiple (USA) | Multitumour | 401 | 63.3 ±11.9 | 0.479 | 6/9 | CES-D (Depression) |
| Quallotine et al., 2017 ^30^ | Baltimore (USA) | Oropharynx | 65 | 59.9 | 0.154 | 6/9 | CES-D (Depression) |
| Ren et al., 2021 ^31^ | Houston (USA) | Multitumour | 505 | 61.5 ±10.6 | 0.224 | 7/9 | CES-D (Depression) |
| Rhoten et al., 2018 ^32^ | Nashville (USA) | Multitumour | 83 | 57.8 ±11.3 | 0.277 | 6/9 | CES-D (Depression) |
| Rogers et al., 2008 ^33^ | Springfield, Illinois (USA) | Multitumour | 58 | 60.4 ±13.1 | 0.259 | 5/9 | CES-D (Depression) |
| Speksnijder et al., 2020 ^34^ | Utrecht (Netherlands) | Oropharynx | 141 | 65.6 ±12.8 | 0.45 | 7/9 | CES-D (Depression) |
| Van Der Meulen et al., 2013 ^35^ | Utrecht (Netherlands) | Multitumour | 91 | 60.7 ±9.8 | 0.297 | 7/9 | CES-D (Depression) |
| Van Wilgen et al., 2004 ^36^ | Groningen (Netherlands) | Multitumour | 155 | 61.3 ±11.9 | 0.33 | 6/9 | CES-D (Depression) |
| Liu et al., 2023 ^37^ | Multiple (Taiwan) | Multitumour | 74439 | N/A | N/A | 6/9 | Suicide (Incidence) |
| Henry et al., 2018-A ^38^ | Montreal (Canada) | Multitumour | 223 | 62.9 ±11.7 | 0.309 | 7/9 | DSM IV Criteria (Anxiety), DSM-IV Criteria (Depression) |
| Gilbert et al., 2011 ^39^ | Nashville (USA) | Multitumour | 94 | 55.4 ±9.7 | 0.16 | 7/9 | DSM-IV Criteria (Depression) |
| McCaffrey et al., 2007 ^40^ | Tampa (USA) | Multitumour | 24 | 73 | 0.167 | 6/9 | DSM-IV Criteria (Depression) |
| Ozsoy et al., 2014 ^41^ | Kayseri (Turkey) | Unknown | 40 | 58.9 ±9.5 | 0 | 5/9 | DSM-IV Criteria (Depression) |
| Hess et al., 2014 ^42^ | Sacramento (USA) | Oropharynx | 162 | 56.9 | 0.12 | 8/9 | DSM-IV Criteria (Depression), DSM IV Criteria (Anxiety) |
| Keszte et al., 2017 ^43^ | Multiple (Germany) | Larynx & Hypopharynx | 176 | N/A | 0.08 | 7/9 | DSM-IV Criteria (Depression), DSM IV Criteria (Anxiety) |
| Kugaya et al., 2000 ^44^ | Kashiwa (Japan) | Multitumour | 107 | 61 ±11.8 | 0.24 | 7/9 | DSM-IV Criteria (Depression), DSM IV Criteria (Anxiety) |
| Sreeraman et al., 2013 ^45^ | Sacramento (USA) | Multitumour | 280 | 60 | N/A | 6/9 | DSM-IV Criteria (Depression), DSM IV Criteria (Anxiety) |
| Lee et al., 2016 ^46^ | Kaohsiung (Taiwan) | Multitumour | 93 | 52.7 ±9.5 | 0.14 | 6/9 | DSM-IV Criteria (Depression), DSM IV Criteria (Anxiety), DSM-IV-Criteria (Insomnia) |
| Unal et al., 2016 ^47^ | Kayseri (Turkey) | Multitumour | 51 | 57.6 ±11.2 | 0.098 | 6/9 | DSM-IV Criteria (Depression), DSM IV Criteria (Anxiety), DSM-IV-Criteria (Insomnia) |
| Li et al., 2020 ^48^ | Taoyuan (Taiwan) | Multitumour | 113 | 52.6 ±10.8 | 0.115 | 7/9 | DSM-IV Criteria (Depression), DSM-V Criteria (Insomnia), DSM-IV Criteria (Post-Trauma) |
| Aminnudin et al., 2020 ^49^ | Sabah (Malaysia) | Oral cavity | 85 | 58.9 ±12.8 | 0.612 | 6/9 | DT (Distress) |
| Chiou et al., 2016 ^50^ | Kaohsiung (Taiwan) | Multitumour | 247 | N/A | N/A | 5/9 | DT (Distress) |
| Wells et al., 2015 ^51^ | Multiple Scotland (United Kingdom) | Multitumour | 280 | 64.5 ±11.4 | 0.27 | 8/9 | DT (Distress) |
| Eades et al., 2013 ^52^ | Montreal (Canada) | Multitumour | 27 | 54.9 ±9.2 | 0.185 | 7/9 | DT (Distress) |
| Ghazali et al., 2017 ^53^ | Liverpool (United Kingdom) | Multitumour | 261 | 63 ±12.4 | 0.32 | 6/9 | DT (Distress) |
| Ghazali et al., 2017 ^54^ | Liverpool (United Kingdom) | Multitumour | 170 | 64.2 ±11.4 | 0.33 | 6/9 | DT (Distress) |
| Kunz et al., 2021 ^55^ | Leipzig (Germany) | Multitumour | 90 | 62.6 ±8.6 | 0.167 | 5/9 | DT (Distress) |
| Lewis et al., 2021 ^56^ | Mumbai (India) | Multitumour | 600 | 51 | 0.183 | 8/9 | DT (Distress) |
| Ma et al., 2013 ^57^ | Ottawa **(Canada)** | Multitumour | 99 | 58.3 ±10.9 | 0.19 | 5/9 | DT (Distress) |
| Matthew et al., 2020 ^58^ | Chennai (India) | Multitumour | 25 | N/A | 0.16 | 5/9 | DT (Distress) |
| Ninu et al., 2016 ^59^ | Florence (Italy) | Multitumour | 86 | 66.78 ±12 | 0.3 | 8/9 | DT (Distress) |
| Patil et al., 2018 ^60^ | Mumbai (India) | Multitumour | 200 | N/A | 0.125 | 6/9 | DT (Distress) |
| Riblet et al., 2014 ^61^ | New Hapshire (USA) | Multitumour | 138 | N/A | N/A | 5/9 | DT (Distress) |
| Schell et al., 2018 ^62^ | Aachen (Germany) | Oral cavity | 100 | 64.4 ±14.7 | 0.43 | 6/9 | DT (Distress) |
| Bultz et al., 2013 ^63^ | Calgary (Canada) | Multitumour | 161 | 61 ±13.06 | 0.244 | 8/9 | Edmonton Symptom Assessment (Depression), Edmonton Symptom Assessment (Anxiety) |
| Duffy et al., 2002 ^64^ | Detroit (USA) | Multitumour | 78 | 61.9 | 0.17 | 7/9 | GDS (Depression) |
| Duffy et al., 2007 ^65^ | Multiple (USA) | Multitumour | 973 | 61 ±10.8 | 0.17 | 6/9 | GDS (Depression) |
| Duffy et al., 2008 ^66^ | Detroit (USA) | Multitumour | 283 | 59.4 ±11.1 | 0.223 | 6/9 | GDS (Depression) |
| Lambert et al., 2005 ^67^ | Detroit (USA) | Multitumour | 385 | 60.3 ±10.8 | 0.27 | 6/9 | GDS (Depression) |
| Shuman et al., 2010 ^68^ | Detroit (USA) | Larynx ± Hypopharynx | 457 | 58.3 ±10.6 | 0.228 | 7/9 | GDS (Depression) |
| Shuman et al., 2012 ^69^ | Multiple (USA) | Multitumour | 559 | 58.4 ±10.7 | 0.225 | 5/9 | GDS (Depression) |
| Thomas et al., 2019 ^70^ | Toronto (Canada) | Multitumour | 274 | 67.8 | 0.248 | 6/9 | GDS (Depression) |
| Bjordal et al., 1995 ^71^ | Oslo (Norway) | Multitumour | 204 | 67 | 0.24 | 6/9 | GHQ-20 (Distress) |
| Aarstad et al., 2014 ^72^ | Bergen (Norway) | Multitumour | 135 | 61 ±10 | 0.244 | 6/9 | GHQ-30 (Distress) |
| Richardson et al., 2017 ^73^ | Auckland (New Zealand) | Multitumour | 28 | N/A | 0.39 | 7/9 | GHQ-HADS-D (Depression)2 (Distress) |
| Adachi et al., 2014 ^74^ | Nagoya (Japan) | Multitumour | 94 | 61.2 ±11.4 | 0.216 | 6/9 | HADS-Combined (Distress) |
| Elaldi et al., 2021 ^75^ | Nice, Caen (France) | Multitumour | 71 | 63.9 ±10.2 | 0.29 | 8/9 | HADS-Combined (Distress) |
| Henry et al., 2014 ^76^ | Montreal (Canada) | Multitumour | 127 | 60.6 ±12.7 | 0.33 | 5/9 | HADS-Combined (Distress) |
| Ichikura et al., 2015 ^77^ | Tokyo (Japan) | Multitumour | 117 | N/A | 0.128 | 5/9 | HADS-Combined (Distress) |
| Krebber et al., 2016 ^78^ | Amsterdam (Netherlands) | Multitumour | 137 | 61.7 ±10.1 | 0.33 | 6/9 | HADS-Combined (Distress) |
| De Leeuw et al., 2007 ^79^ | Amsterdam (Netherlands) | Multitumour | 45 | 61 | 0.42 | 3/9 | HADS-Combined (Distress) |
| Singer et al., 2012 ^80^ | Leipzig (Germany) | Multitumour | 113 | 58 | 0.2 | 7/9 | HADS-Combined (Distress) |
| Wang et al., 2019 ^81^ | Shanghai (China) | Multitumour | 211 | 62.1 ±8.2 | 0.009 | 6/9 | HADS-Combined (Distress) |
| Cohen et al., 2018 ^82^ | Montreal (Canada) | Multitumour | 84 | 63.3 ±9.17 | 0.31 | 7/9 | HADS-Combined (Distress), DSM-IV Criteria (Depression), DSM IV Criteria (Anxiety) |
| Van Beek et al., 2022 ^83^ | Multiple (Netherlands) | Multitumour | 558 | 64 ±9 | 0.26 | 8/9 | HADS-Combined (Distress), HADS-D (Depression), HADS-A (Anxiety) |
| De Leeuw et al., 2009 ^84^ | Amsterdam (Netherlands) | Multitumour | 55 | 63 | 0.31 | 6/9 | HADS-Combined (Distress), HADS-D (Depression), HADS-A (Anxiety) |
| Allison et al., 2004 ^85^ | Montreal (Canada) | Multitumour | 49 | N/A | N/A | 5/9 | HADS-D (Depression) |
| Chang et al., 2017 ^86^ | Chiayi (Taiwan) | Oral cavity | 184 | N/A | N/A | 7/9 | HADS-D (Depression) |
| Chen et al., 2018 ^87^ | Taoyuan (Taiwan) | Oral cavity | 151 | 52.3 ±8.6 | 0.126 | 8/9 | HADS-D (Depression) |
| Chen et al., 2019 ^88^ | Taoyuan (Taiwan) | Multitumour | 50 | 49.1 | 0.026 | 7/9 | HADS-D (Depression) |
| Eadie et al., 2018 ^89^ | Seattle (USA) | Multitumour | 88 | 66 ±9 | 0.34 | 7/9 | HADS-D (Depression) |
| Hammerlid et al., 1997 ^90^ | Gothenburg (Sweden) | Multitumour | 42 | 64.3 | N/A | 6/9 | HADS-D (Depression) |
| Hartl et al., 2009 ^91^ | Villejuif (France) | Oral cavity | 12 | 51 | 0.16 | 5/9 | HADS-D (Depression) |
| Lee et al., 2015 ^92^ | Taoyuan (Taiwan) | Multitumour | 104 | 54.8 ±14 | 0.077 | 7/9 | HADS-D (Depression) |
| Lee-Preston et al., 2004 ^93^ | Newcastle upon Tyne (United Kingdom) | Larynx & Hypopharynx | 36 | 67 | 0.19 | 8/9 | HADS-D (Depression) |
| Patterson et al., 2021 ^94^ | Multiple (United Kingdom) | Multitumour | 2561 | N/A | 0.286 | 8/9 | HADS-D (Depression) |
| Wang et al., 2021 ^95^ | Fuzhou (China) | Nasopharynx | 232 | 51 | 0.267 | 6/9 | HADS-D (Depression) |
| Chen et al., 2012 ^96^ | Taoyuan (Taiwan) | Oral cavity | 82 | N/A | 0.024 | 7/9 | HADS-D (Depression) |
| Hassanein et al., 2005 ^97^ | Manchester (United Kingdom) | Multitumour | 68 | 58 | 0.31 | 7/9 | HADS-D (Depression) |
| Singer et al., 2005 ^98^ | Multiple (Germany) | Larynx & Hypopharynx | 189 | 64 | 0.07 | 7/9 | HADS-D (Depression) |
| Aghajanzadeh et al., 2020 ^99^ | Gothenburg (Sweden) | Multitumour | 50 | 58 | 0.38 | 6/9 | HADS-D (Depression), HADS-A (Anxiety) |
| Airoldi et al., 2011 ^100^ | Turin (Italy) | Oral cavity | 33 | 60.5 ±11 | 0.28 | 8/9 | HADS-D (Depression), HADS-A (Anxiety) |
| Almstahl et al., 2019 ^101^ | Gothenburg (Sweden) | Multitumour | 29 | 59 ±8 | 0.34 | 5/9 | HADS-D (Depression), HADS-A (Anxiety) |
| Van Beek et al., 2020 ^102^ | Amsterdam (Netherlands) | Multitumour | 170 | 61 ±9 | 0.27 | 7/9 | HADS-D (Depression), HADS-A (Anxiety) |
| Berg et al., 2023 ^103^ | Gothenburg (Sweden) | Multitumour | 165 | 64.2 ±8.1 | 0.364 | 7/9 | HADS-D (Depression), HADS-A (Anxiety) |
| Bernstein et al., 2018 ^104^ | Toronto (Canada) | Multitumour | 80 | 58.3 ±7.6 | 0.15 | 8/9 | HADS-D (Depression), HADS-A (Anxiety) |
| Chaillou et al., 2019 ^105^ | Lille (France) | Multitumour | 34 | 60.5 | 0.27 | 5/9 | HADS-D (Depression), HADS-A (Anxiety) |
| Chen et al., 2009-B ^106^ | Sacramento (USA) | Multitumour | 40 | 59.5 ±15 | 0.38 | 8/9 | HADS-D (Depression), HADS-A (Anxiety) |
| Chen et al., 2020 ^107^ | Taoyuan (Taiwan) | Multitumour | 150 | 63.1 ±10.6 | 0.06 | 6/9 | HADS-D (Depression), HADS-A (Anxiety) |
| D'Souza et al., 2013 ^108^ | Montreal (Canada) | Multitumour | 53 | 61.7 ±14.6 | 0.208 | 6/9 | HADS-D (Depression), HADS-A (Anxiety) |
| Ehrsson et al., 2021 ^109^ | Uppsala (Sweden) | Multitumour | 273 | 63 ±11 | 0.275 | 7/9 | HADS-D (Depression), HADS-A (Anxiety) |
| Elani et al., 2011 ^110^ | Montreal (Canada) | Multitumour | 157 | 62.6 ±11.8 | 0.29 | 7/9 | HADS-D (Depression), HADS-A (Anxiety) |
| Finizia et al., 1998 ^111^ | Gothenburg (Sweden) | Multitumour | 28 | 65 | 0.14 | 7/9 | HADS-D (Depression), HADS-A (Anxiety) |
| Finizia et al., 2001-A ^112^ | Gothenburg (Sweden) | Larynx & Hypopharynx | 89 | 70 | 0.13 | 6/9 | HADS-D (Depression), HADS-A (Anxiety) |
| Gosak et al., 2020 ^113^ | Ljubljana (Slovenia) | Multitumour | 40 | 62.5 ±7.61 | 0 | 6/9 | HADS-D (Depression), HADS-A (Anxiety) |
| Griffiths et al., 1999 ^114^ | London (United Kingdom) | Unknown | 615 | N/A | N/A | 7/9 | HADS-D (Depression), HADS-A (Anxiety) |
| Hammerlid et al., 1996 ^115^ | Gothenburg (Sweden) | Multitumour | 79 | 59 | 0.33 | 7/9 | HADS-D (Depression), HADS-A (Anxiety) |
| Hammerlid et al., 1999 ^116^ | Gothenburg, Malmo, Lund, Oslo (Sweden, Norway) | Multitumour | 290 | 63 | 0.28 | 7/9 | HADS-D (Depression), HADS-A (Anxiety) |
| Hammerlid et al., 2001 ^117^ | Gothenburg (Sweden) | Multitumour | 232 | 61 | 0.3 | 7/9 | HADS-D (Depression), HADS-A (Anxiety) |
| Horney et al., 2011 ^118^ | Multiple, Southeast England (United Kingdom) | Multitumour | 103 | 63 ±13.9 | 0.29 | 8/9 | HADS-D (Depression), HADS-A (Anxiety) |
| Hutton et al., 2001 ^119^ | London (United Kingdom) | Multitumour | 9 | 68 | 0.22 | 3/9 | HADS-D (Depression), HADS-A (Anxiety) |
| Jenewein et al., 2007 ^120^ | Zurich (Switzerland) | Oral cavity | 31 | 58.2 ±10.1 | 0 | 6/9 | HADS-D (Depression), HADS-A (Anxiety) |
| Johansson et al., 2008 ^121^ | Gothenburg (Sweden) | Larynx & Hypopharynx | 100 | 67 | 0.17 | 7/9 | HADS-D (Depression), HADS-A (Anxiety) |
| Kelly et al., 2007 ^122^ | Newcastle upon Tyne (United Kingdom) | Multitumour | 110 | N/A | 0.269 | 6/9 | HADS-D (Depression), HADS-A (Anxiety) |
| Krebbers et al., 2021 ^123^ | Maastricht (Netherlands) | Multitumour | 84 | 65.8 ±10 | 0.31 | 7/9 | HADS-D (Depression), HADS-A (Anxiety) |
| Liu et al., 2013 ^124^ | Shanghai (China) | Paranasal sinus | 61 | 50 | 0.28 | 6/9 | HADS-D (Depression), HADS-A (Anxiety) |
| McCarter et al., 2022 ^125^ | Callahan (Australia) | Multitumour | 98 | 59 ±0.2 | N/A | 7/9 | HADS-D (Depression), HADS-A (Anxiety) |
| McDowell et al., 2018 ^126^ | Toronto (Canada) | Nasopharynx | 107 | 56.1 ±10 | 0.36 | 9/9 | HADS-D (Depression), HADS-A (Anxiety) |
| Neilson et al., 2013 ^127^ | Melbourne (Australia) | Multitumour | 101 | 63 | 0.16 | 7/9 | HADS-D (Depression), HADS-A (Anxiety) |
| Nikoloudi et al., 2020 ^128^ | Athens (Greece) | Multitumour | 55 | 61.35 ±14.37 | 0.35 | 7/9 | HADS-D (Depression), HADS-A (Anxiety) |
| Offerman et al., 2010 ^129^ | Rotterdam (Netherlands) | Multitumour | 20 | 60.7 ±10.37 | 0 | 5/9 | HADS-D (Depression), HADS-A (Anxiety) |
| Pandey et al., 2007 ^130^ | Varanasi (India) | Multitumour | 123 | 54.3 ±11.8 | 0.244 | 6/9 | HADS-D (Depression), HADS-A (Anxiety) |
| Pauli et al., 2012 ^131^ | Gothenburg (Sweden) | Multitumour | 75 | 62 | 0.4 | 7/9 | HADS-D (Depression), HADS-A (Anxiety) |
| Petruson et al., 2002 ^132^ | Gothenburg (Sweden) | Multitumour | 92 | 61 | 0.27 | 7/9 | HADS-D (Depression), HADS-A (Anxiety) |
| Petruson et al., 2005 ^133^ | Gothenburg (Sweden) | Multitumour | 49 | 60 | 0.2 | 6/9 | HADS-D (Depression), HADS-A (Anxiety) |
| Rampling et al., 2003 ^134^ | Manchester (United Kingdom) | Multitumour | 92 | 61.5 | 0.2 | 6/9 | HADS-D (Depression), HADS-A (Anxiety) |
| Rogers et al., 2006 ^135^ | Liverpool (United Kingdom) | Multitumour | 197 | N/A | 0.44 | 6/9 | HADS-D (Depression), HADS-A (Anxiety) |
| Rose et al., 2001 ^136^ | Brisbane **(Australia)** | Multitumour | 58 | N/A | 0.293 | 5/9 | HADS-D (Depression), HADS-A (Anxiety) |
| Singer et al., 2009 ^137^ | Leipzig, Dresden (Germany) | Larynx & Hypopharynx | 206 | 0.068 | N/A | 7/9 | HADS-D (Depression), HADS-A (Anxiety) |
| So et al., 2020 ^138^ | Montreal (Canada) | Nasopharynx | 73 | 52 ±8 | 0.71 | 8/9 | HADS-D (Depression), HADS-A (Anxiety) |
| Sunderland et al., 2022 ^139^ | Northland (New Zealand) | Multitumour | 110 | 65 | 0.32 | 6/9 | HADS-D (Depression), HADS-A (Anxiety) |
| Suzuki et al., 2016 ^140^ | Tokyo (Japan) | Multitumour | 194 | 64 ±12.5 | 0.392 | 6/9 | HADS-D (Depression), HADS-A (Anxiety) |
| Tang et al., 2023 ^141^ | Nanjin (China) | Multitumour | 85 | N/A | 0.318 | 7/9 | HADS-D (Depression), HADS-A (Anxiety) |
| Veer et al., 2010 ^142^ | Romford (United Kingdom) | Multitumour | 106 | N/A | N/A | 4/9 | HADS-D (Depression), HADS-A (Anxiety) |
| Wulff et al., 2021 ^143^ | Multiple (Denmarck & Sweeden) | Larynx & Hypopharynx | 172 | 71.4 ±9.9 | 0.15 | 8/9 | HADS-D (Depression), HADS-A (Anxiety) |
| Zahid et al., 2021 ^144^ | Karachi (Karachi) | Multitumour | 250 | 51.6 | 0.21 | 7/9 | HADS-D (Depression), HADS-A (Anxiety) |
| Zwahlen et al., 2008 ^145^ | Hong Kong (China) | Oral cavity | 31 | 58.2 ±10.1 | 0 | 6/9 | HADS-D (Depression), HADS-A (Anxiety) |
| Schiefke et al., 2009 ^146^ | Leipzig, Berlin (Germany) | Multitumour | 49 | 63 | 0.24 | 7/9 | HADS-D (Depression), HADS-A (Anxiety) |
| Shiraz et al., 2014 ^147^ | London (United Kingdom) | Multitumour | 101 | N/A | 0.39 | 5/9 | HADS-D (Depression), HADS-A (Anxiety), Acute Stress Disorder Scale (Distress) |
| Posluszny et al., 2015 ^148^ | Pittsburgh (USA) | Multitumour | 42 | 55 ±8.7 | 0.24 | 8/9 | HADS-D (Depression), HADS-A (Anxiety), DSM-IV Criteria (Post-Trauma) |
| Ghiggia et al., 2016 ^149^ | Turin (Italy) | Nasopharynx | 21 | 54.1 ±12 | 0.143 | 7/9 | HADS-D (Depression), HADS-A (Anxiety), DT (Distress) |
| Rodrigues-Oliveira et al., 2021 ^150^ | Sao Paulo (Brazil) | Multitumour | 50 | 58.8 ±9.89 | 0.22 | 8/9 | HADS-D (Depression), HADS-A (Anxiety), DT (Distress) |
| Joseph et al., 2013 ^151^ | Manchester (United Kingdom) | Multitumour | 220 | 59.5 | 0.186 | 6/9 | HADS-D (Depression), HADS-A (Anxiety), HADS-Combined (Distress) |
| Van Nieuwenhuizen et al., 2014 ^152^ | Amsterdam (Netherlands) | Multitumour | 11 | 62 ±8 | 0.64 | 6/9 | HADS-D (Depression), HADS-A (Anxiety), HADS-Combined (Distress) |
| Verdonck-deLeeuw et al., 2010 ^153^ | Amsterdam (Netherlands) | Multitumour | 85 | 59 | 0.36 | 7/9 | HADS-D (Depression), HADS-A (Anxiety), HADS-Combined (Distress) |
| Bozec et al., 2021 ^154^ | Nice (France) | Multitumour | 48 | N/A | 0.25 | 7/9 | HADS-D (Depression), HADS-A (Anxiety), HADS-Combined (Distress) |
| Budhrani-Shani et al., 2018 ^155^ | Boston (USA) | Multitumour | 30 | N/A | 0 | 5/9 | HADS-D (Depression), HADS-A (Anxiety), PSQI |
| Bornbaum et al., 2011 ^156^ | London (United Kingdom) | Multitumour | 49 | 60.1 | 0.31 | 7/9 | HADS-D (Depression), HADS-A (Anxiety)+BNHADS-D (Depression)24 |
| Bozec et al., 2020 ^157^ | Multiple (France) | Multitumour | 64 | 78.5 ±4.6 | 0.41 | 6/9 | HADS-D (Depression), HADS-Combined (Distress), HADS-A (Anxiety) |
| Chen et al., 2009-A ^158^ | Taoyuan (Taiwan) | Oral cavity | 112 | 53.4 ±10.53 | 0.036 | 7/9 | HADS-D (Depression),HADS-A (Anxiety) |
| Hong et al., 2013 ^159^ | Fuzhou (China) | Multitumour | 226 | N/A | N/A | 6/9 | HADS-D (Depression),HADS-A (Anxiety) |
| Lazure et al., 2009 ^160^ | Nebraska (USA) | Multitumour | 36 | 62.5 ±10.1 | 0.57 | 5/9 | Hamilton Depression Scale (Depression) |
| Riedl et al., 2018 ^161^ | Insbruck (Austria) | Multitumour | 49 | N/A | N/A | 6/9 | Hornheide (Distress) |
| Sun et al., 2020 | Multiple (Taiwan) | Multitumour | 66931 | 53.7 ±13.4 | 0.125 | 7/9 | ICD-9 Criteria (Anxiety) |
| Lawrence et al., 2017 ^162^ | Multiple (USA) | Multitumour | 36420 | 65.7 | 0.26 | 6/9 | ICD-9 Criteria (Depression) |
| LI et al., 2015 ^163^ | Multiple (China) | Multitumour | 921 | N/A | 0.212 | 6/9 | ICD-9 Criteria (Depression) |
| Rieke et al., 2016 ^164^ | Multiple (USA) | Multitumour | 3157 | N/A | 0.32 | 6/9 | ICD-9 Criteria (Depression) |
| Rohde et al., 2018 ^165^ | Multiple (USA) | Multitumour | 71541 | 62.2 ±13.5 | 0.282 | 6/9 | ICD-9 Criteria (Depression) |
| Rieke et al., 2017 ^166^ | Multiple (USA) | Multitumour | 3476 | N/A | 0.34 | 5/9 | ICD-9 Criteria (Depression), HADS-A (Anxiety), DSM-IV Criteria (Post-Trauma), HADS-D (Depression) |
| Bigelow et al., 2020 ^167^ | Multiple (USA) | Oropharynx | 2497 | 72 | 0.254 | 6/9 | ICD-9 Criteria (Depression), ICD-9 Criteria (Anxiety) |
| Jeffery et al., 2019 ^168^ | Multiple (USA) | Multitumour | 2994 | N/A | 0.306 | 5/9 | ICD-9 Criteria (Depression), ICD-9 Criteria (Anxiety) |
| Madrigal et al., 2023 ^169^ | Multiple (USA) | Multitumour | 133,018 | 63 | 0.317 | 6/9 | ICD-9 Criteria (Depression), ICD-9 Criteria (Anxiety) |
| Mirosevic et al., 2019 ^170^ | Multiple (Netherlands) | Multitumour | 178 | 62.3 ±9.7 | 0.306 | 6/9 | ICD-9 Criteria (Depression), ICD-9 Criteria (Anxiety) |
| Mukherjee et al., 2022 ^171^ | Kolkata (India) | Multitumour | 771 | 54.9 ±13.2 | 0.28 | 6/9 | ICD-9 Criteria (Depression), ICD-HADS-D (Depression)0 Criteria (Anxiety) |
| Wang et al., 2023 ^172^ | Cleveland (USA) | Multitumour | 22 | N/A | N/A | 6/9 | IES-R (Post-Trauma) |
| Choi et al., 2020 ^173^ | Multiple (South Korea) | Multitumour | 9000 | N/A | N/A | 6/9 | Incidence rate (Suicide) |
| Osazuwa-Peters et al., 2018 ^174^ | Multiple (USA) | Multitumour | 287901 | N/A | 0.29 | 7/9 | Incidence rate (Suicide) |
| Nugent et al., 2021 ^175^ | Multiple (USA) | Multitumour | 7803 | 64.6 ±10.7 | 0.015 | 6/9 | Incidence rate (Suicide), ICD-9 Criteria (Anxiety), ICD-9 Criteria (Depression) |
| Savard et al., 2009 ^176^ | Quebec (Canada) | Multitumour | 23 | N/A | N/A | 6/9 | Insomnia interview scale |
| Rapoport et al., 1993 ^177^ | Tel Aviv (Israel) | Multitumour | 55 | 60.7 ±15 | 0.27 | 6/9 | MAACL (Depression), STPI Form X-2 (Anxiety) |
| Hajdu et al., 2021 ^178^ | Multiple (Denmark) | Multitumour | 235 | 63 ±9 | 0.16 | 6/9 | Major Depression Index (Depression),HADS-A (Anxiety), 3 |
| Fujii et al., 2001 ^179^ | Tokyo (Japan) | Multitumour | 41 | 59.6 | 0.2 | 5/9 | Manifest Anxiety Scale (Anxiety) |
| Yadav et al., 2019 ^180^ | Karnakata (India) | Multitumour | 100 | 50 ±11.8 | 0.36 | 5/9 | Mini-5 Criteria (Depression) |
| Moschopoulou et al., 2018 ^181^ | London (United Kingdom) | Multitumour | 93 | 66 ±11 | 0.42 | 7/9 | PCL-C (Post-Trauma) |
| Britton et al., 2018 ^182^ | Multiple (Australia) | Multitumour | 150 | 58 ±10 | 0.17 | 7/9 | PHQ-9 (Depression) |
| Hammermüller et al., 2021 ^183^ | Leipzig (Germany) | Multitumour | 817 | 62.7 ±10.4 | 0.228 | 6/9 | PHQ-9 (Depression) |
| Manne et al., 2022 ^184^ | New Jersey & California (USA) | Multitumour | 232 | 62.5 ±10.5 | 0.293 | 7/9 | PHQ-9 (Depression) |
| McCarter et al., 2018 ^185^ | Newcastle (Australia) | Multitumour | 307 | 58 ±10 | 0.21 | 7/9 | PHQ-9 (Depression) |
| Omoro et al., 2006 ^186^ | Nairobi (Kenya) | Multitumour | 48 | 55.7 ±12 | 0.08 | 7/9 | PHQ-9 (Depression) |
| Nitin et al., 2019 ^187^ | Karnakata (India) | Multitumour | 70 | 55.1 ±11.3 | 0.257 | 6/9 | PHQ-9 (Depression) |
| Shinn et al., 2016 ^188^ | Houston (USA) | Oropharynx | 130 | 56.3 ±9.6 | 0.169 | 7/9 | PHQ-9 (Depression) |
| Torrealba et al., 2022 ^189^ | Paraná (Brazil) | Multitumour | 60 | 60.6 ±10.9 | 0.388 | 7/9 | PHQ-9 (Depression) |
| Eastburn et al., 2022 ^190^ | Pittsburgh (USA) | Multitumour | 228 | 64.95 ±10.2 | 0.21 | 7/9 | PHQ-9 (Depression), GAD-7 (Anxiety) |
| Gascon et al., 2022 ^191^ | Toronto (Canada) | Multitumour | 347 | 60.7 ±12.3 | 0.233 | 5/9 | PHQ-9 (Depression), GAD-7 (Anxiety) |
| Macias et al., 2021 ^192^ | Multiple (USA) | Multitumour | 250 | 62.3 ±11.9 | 0.296 | 7/9 | PHQ-9 (Depression), GAD-7 (Anxiety) |
| Li et al., 2017 ^193^ | Tokyo (Japan) | Multitumour | 77 | N/A | 0.37 | 7/9 | PSQI |
| Santoso et al., 2021 ^194^ | Multiple (Netherlands) | Multitumour | 560 | 63 ±9 | 0.25 | 7/9 | PSQI |
| Richardson et al., 2016 ^195^ | Auckland (New Zealand) | Multitumour | 65 | N/A | 0.28 | 5/9 | PSS-SR (Post-Trauma) |
| Barber et al., 2015 ^196^ | Alberta (Canada) | Multitumour | 71 | 59.7 | 0.296 | 6/9 | QIDS-SR (Depression) |
| Katz et al., 2004 ^197^ | Ontario (Canada) | Multitumour | 60 | 60.5 ±13 | 0.22 | 7/9 | SDS (Depression) |
| Sehlen et al., 2003 ^198^ | Munich (Germany) | Multitumour | 121 | 58.1 | 0.198 | 4/9 | SDS (Depression) |
| Liu et al., 2022 ^199^ | Zhuhai (China) | Multitumour | 257 | 52 | 0.338 | 6/9 | SDS (Depression), SAS (Anxiety) |
| Lou et al., 2020 ^200^ | Jiangsu (China) | Multitumour | 58 | 46 ±8.3 | 0.4 | 6/9 | SDS (Depression), Self-Rating Anxiety Scale (Anxiety) |
| Firmeza et al., 2017 ^201^ | Fortaleza (Brazil) | Multitumour | 20 | N/A | N/A | 4/9 | STAI (Anxiety) |
| Chang et al., 2022 ^202^ | Taoyuan (Taiwan) | Multitumour | 114 | N/A | 0.079 | 6/9 | State Anxiety Inventory (Anxiety) |
| Jehn et al., 2022 ^203^ | Multiple (Germany, Switzerland) | Oral cavity | 1234 | N/A | 0.248 | 7/9 | STATE-TRAIT (Anxiety), DS (Depression) |
| Chang et al., 2019 ^204^ | Changhua (Taiwan) | Multitumour | 286 | 57 ±11.86 | 0.04 | 7/9 | Suicidal Ideation, DSM-IV Criteria (Depression) |
| Tseng et al., 2022 ^205^ | Taoyuan (Taiwan) | Multitumour | 153 | 56.9 ±9.5 | 0.046 | 7/9 | Suicidal Ideation, DSM-IV-Criteria (Insomnia), DSM-IV Criteria (Depression) |
| Hadas et al., 2022 ^206^ | Munich (Germany) | Multitumour | 453 | 64.54 | 0.28 | 6/9 | WHO-5 (Depression) |

**Supplementary Table 5**: **Suicide incidence of the included studies**

| **Article** | **Consumed Suicides** | **Total Person-Time at Risk (year)** | **Suicide Incidence** |
| --- | --- | --- | --- |
| Osazuwa-Peters et al, 2018 | 1036 | 1646032 | 63 |
| Choi et al, 2020 | 22 | 9007,33 | 244,4 |
| Liu et al, 2023 | 396 | 296515 | 133,52 |
| Nugent et al, 2021 | 51 | 25015 | 203,95 |

**Supplementary**
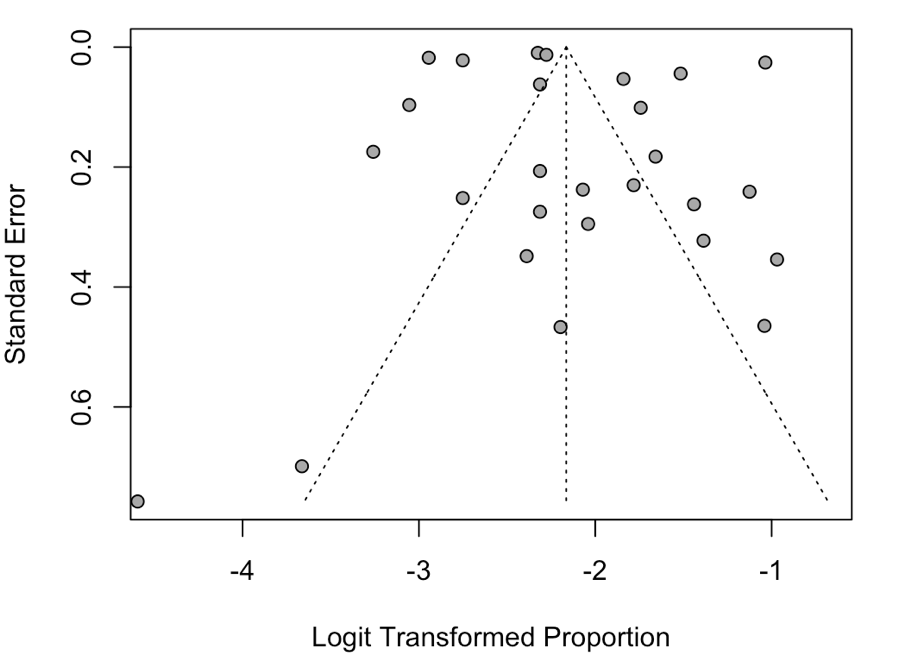
**Figure 1: Funnel plots for publication bias**


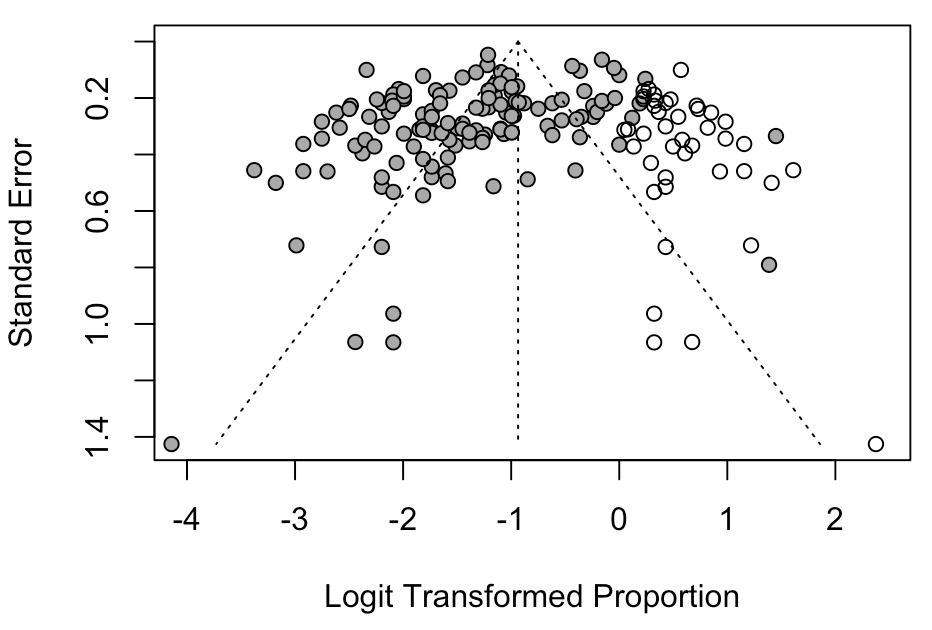


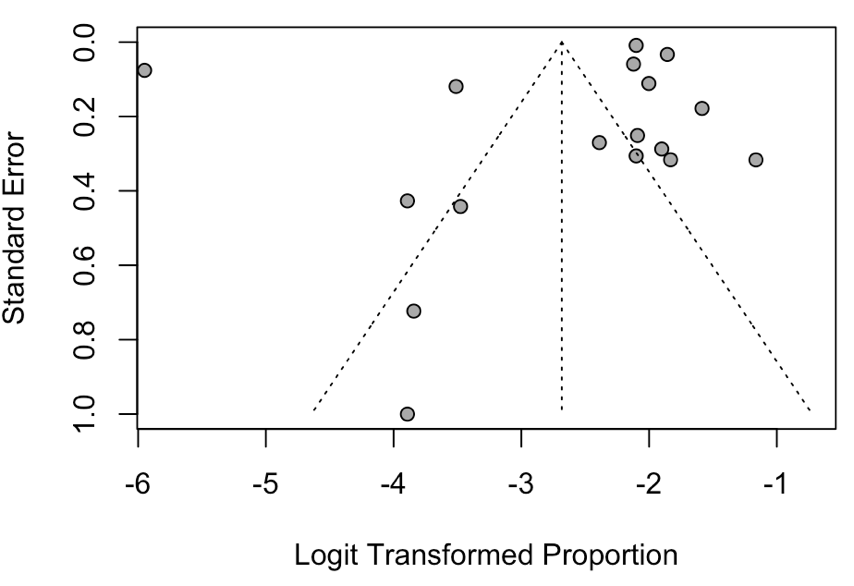

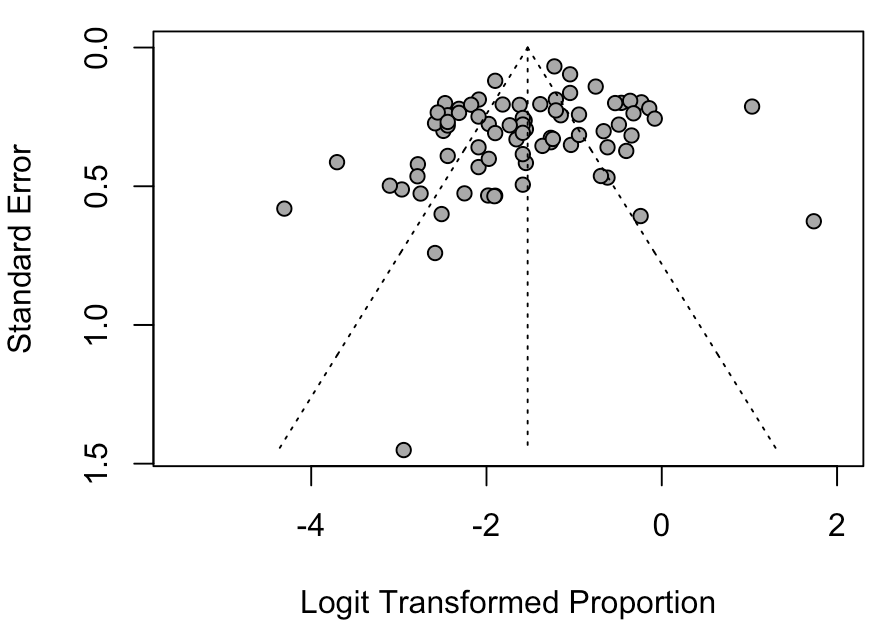


**Supplementary Figure 1-C**. Proportion of patients with clinically significant anxiety symptoms.

**Supplementary Figure 1-A**. Proportion of patients with clinically significant depressive symptoms (articles added by trim and fill method correction are represented in white).

**Supplementary Figure 1-B**. Proportion of patients with depressive disorders.

**Supplementary Figure 1-D**. Proportion of patients with anxiety disorders.


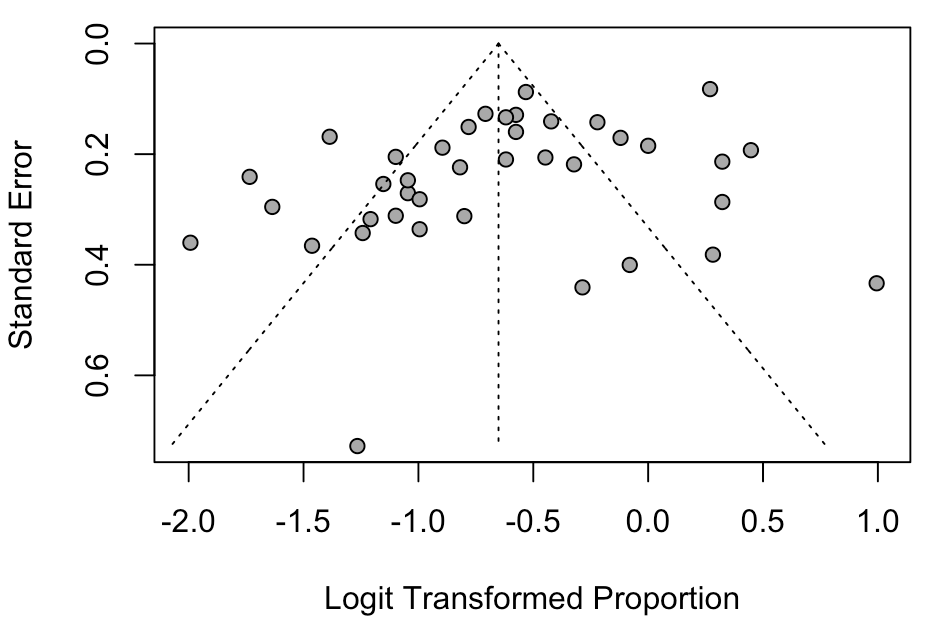


**Supplementary Figure 1-E**. Proportion of patients with clinically significant distress.

**Supplementary Table 6: Meta-regressions for the studied outcomes**

| **Meta-regression** | | **No. of**  **Studies** | **β Coefficient** | **SE** | **95% CI** | | **P value** |
| --- | --- | --- | --- | --- | --- | --- | --- |
| **Depressive Symptoms** | | | | | | | |
| **NOS Scale** | | 137 | -0.1786 | 0.0738 | -0.325 | 0.013 | 0.05 |
| **Publication year** | | 137 | -0.0141 | 0.0095 | -0.033 | 0.005 | 0.14 |
| **Mean age** | | 116 | -0.0084 | 0.0107 | -0.030 | 0.013 | 0.43 |
| **% Female** | | 128 | 0.8709 | 0.6449 | -0.405 | 2.147 | 0.20 |
| **% Alcohol use** | | 40 | -0.6018 | 0.6806 | -1.980 | 0.780 | 0.38 |
| **% Tobacco use** | | 51 | 0.2042 | 0.4841 | -0.769 | 1.177 | 0.68 |
| **% Low education** | | 55 | -0.0527 | 0.6070 | -1.270 | 1.165 | 0.93 |
| **% High education** | | 55 | -0.0318 | 0.6134 | -1.262 | 1.199 | 0.96 |
| **% White** | | 33 | -0.2427 | 0.7569 | -1.786 | 1.301 | 0.75 |
| **% Employed** | | 53 | 0.0777 | 0.6471 | -1.221 | 1.377 | 0.90 |
| **% Married** | | 84 | 0.0132 | 0.6451 | -1.270 | 1.297 | 0.98 |
| **% Recurrence** | | 24 | -1.7270 | 2.7630 | -7.457 | 4.003 | 0.54 |
| **% Larynx & Hypopharynx** | | 101 | -0.1047 | 0.3101 | -0.720 | 0.511 | 0.74 |
| **% Oropharynx** | | 82 | 0.2421 | 0.3138 | -0.382 | 0.867 | 0.44 |
| **% Oral cavity** | | 105 | -0.4981 | 0.2736 | -1.041 | 0.045 | 0.07 |
| **% Nasopharynx** | | 53 | 0.5403 | 0.4432 | -0.349 | 1.430 | 0.23 |
| **% Initial stage (I, II)** | | 91 | 0.3201 | 0.4381 | -0.5504 | 1.191 | 0.47 |
| **% Advanced stage (III, IV)** | | 93 | -0.2619 | 0.3978 | -1.052 | 0.528 | 0.51 |
| **% Metastatic** | | 49 | 1.4506 | 1.4391 | -1.445 | 4.346 | 0.32 |
| **% Positive HPV** | | 9 | 1.2190 | 1.5121 | -2.357 | 4.795 | 0.45 |
| **Months since diagnosis** | | 18 | 0.0092 | 0.0056 | -0.003 | 0.021 | 0.12 |
| **% Surgery (total)** | | 91 | -0.3866 | 0.2583 | -0.900 | 0.127 | 0.14 |
| **% Only surgery** | | 81 | -0.1294 | 0.4018 | -0.929 | 0.670 | 0.75 |
| **% Multimodal surgery** | | 74 | -0.6695 | 0.3418 | -1.351 | 0.012 | 0.05 |
| **% RT (total)** | | 66 | 0.4704 | 0.5557 | -0.640 | 1.581 | 0.40 |
| **% Only RT** | | 102 | -0.0468 | 0.3640 | -0.769 | 0.676 | 0.90 |
| **% (Neo)Adjuvant CT** | | 60 | 0.1094 | 0.4366 | -0.765 | 0.9833 | 0.80 |
| **% Primary CCRT** | | 65 | 0.5017 | 0.3436 | -0.185 | 1.188 | 0.15 |
| **% Laryngectomy** | | 36 | -0.2869 | 0.3873 | -1.074 | 0.500 | 0.46 |
| **% Pain** | | 11 | -3.3760 | 2.0276 | -7.963 | 1.211 | 0.13 |
| **Depressive Disorders** | | | | | | | |
| **NOS Scale** | | 27 | -0.1047 | 0.2009 | -0.519 | 0.310 | 0.61 |
| **Publication year** | | 27 | -0.0175 | 0.0277 | -0.075 | 0.400 | 0.53 |
| **Mean age** | | 22 | -0.0197 | 0.0265 | -0.075 | 0.036 | 0.47 |
| **% Female** | | 26 | -1.1451 | 1.3517 | -3.935 | 1.645 | 0.41 |
| **% Alcohol use** | | 13 | 0.0099 | 0.7630 | -1.669 | 1.689 | 0.99 |
| **% Tobacco use** | | 13 | 0.2118 | 0.6513 | -1.222 | 1.645 | 0.75 |
| **% Low education** | | 7 | -0.7219 | 2.9879 | -8.400 | 6.956 | 0.82 |
| **% White** | | 7 | 0.5240 | 2.2792 | -5.335 | 6.383 | 0.83 |
| **% Married** | | 14 | -1.6531 | 0.9308 | -3.681 | 0.375 | 0.10 |
| **% Larynx & Hypopharynx** | | 18 | -1.1327 | 0.9433 | -3.133 | 0.867 | 0.25 |
| **% Oropharynx** | | 14 | 0.0588 | 0.6655 | -1.391 | 1.509 | 0.93 |
| **% Oral cavity** | | 18 | 0.6811 | 0.8433 | -1.107 | 2.469 | 0.43 |
| **% Nasopharynx** | | 9 | 11.5907 | 5.5872 | -1.618 | 24.800 | 0.08 |
| **% Initial stage (I, II)** | | 18 | -1.4829 | 1.1411 | -3.901 | 0.937 | 0.21 |
| **% Advanced stage (III, IV)** | | 20 | 1.3933 | 0.9820 | -0.670 | 3.457 | 0.17 |
| **% Metastatic** | | 8 | 0.7402 | 3.3286 | -7.405 | 8.885 | 0.83 |
| **% Surgery (total)** | | 11 | -0.2203 | 1.0038 | -2.491 | 2.051 | 0.83 |
| **% RT (total)** | | 14 | 1.3996 | 1.0659 | -0.923 | 3.722 | 0.21 |
| **% Only RT** | | 7 | 0.3224 | 1.4764 | -3.473 | 4.118 | 0.84 |
| **Anxiety Symptoms** | | | | | | | |
| **NOS Scale** | 78 | | -0.1795 | 0.1147 | -0.408 | 0.049 | 0.12 |
| **Publication year** | 78 | | 0.0189 | 0.0161 | -0.013 | 0.051 | 0.24 |
| **Mean age** | 64 | | -0.0129 | 0.0132 | -0.039 | 0.013 | 0.33 |
| **% Female** | 71 | | 1.2848 | 0.8938 | -0.498 | 3.068 | 0.16 |
| **% Alcohol use** | 16 | | 1.1587 | 0.5697 | -0.063 | 2.381 | 0.06 |
| **% Tobacco use** | 27 | | 0.3481 | 0.5069 | -0.696 | 1.392 | 0.50 |
| **% Low education** | 38 | | -0.9861 | 0.9768 | -2.967 | 0.995 | 0.32 |
| **% High education** | 32 | | 1.5210 | 1.0306 | -0.5838 | 3.6259 | 0.15 |
| **% White** | 13 | | -1.5328 | 0.6892 | -3.050 | 0.016 | 0.05 |
| **% Employed** | 33 | | 0.8573 | 1.0036 | -1.190 | 2.904 | 0.40 |
| **% Married** | 49 | | 0.9305 | 0.9327 | -0.946 | 2.807 | 0.99 |
| **% Larynx & Hypopharynx** | 51 | | -0.7566 | 0.3976 | -1.556 | 0.042 | 0.06 |
| **% Oropharynx** | 42 | | -0.3092 | 0.4846 | 0.527 | -1.289 | 0.40 |
| **% Oral cavity** | 55 | | -0.1825 | 0.3951 | -0.975 | 0.610 | 0.65 |
| **% Nasopharynx** | 25 | | 1.0186 | 0.4991 | 0.053 | -0.014 | 0.23 |
| **% Initial stage (I, II)** | 46 | | -1.0232 | 0.6038 | -2.240 | 0.194 | 0.09 |
| **% Advanced stage (III, IV)** | 52 | | 0.5993 | 0.5212 | -0.448 | 1.646 | 0.26 |
| **% Metastatic** | 31 | | 2.2415 | 2.5183 | -2.909 | 7.392 | 0.38 |
| **Months since diagnosis** | 12 | | 0.0055 | 0.0070 | 0.010 | 0.021 | 0.45 |
| **% Surgery (total)** | 49 | | -0.6850 | 0.3391 | -1.367 | -0.003 | 0.04* |
| **% Only surgery** | 49 | | 0.1243 | 0.4223 | -0.725 | 0.974 | 0.77 |
| **% Multimodal surgery** | 44 | | -0.629 | 0.475 | -1.588 | 0.330 | 0.19 |
| **% RT (total)** | 58 | | 0.1226 | 0.4884 | -0.856 | 1.101 | 0.80 |
| **% Only RT** | 40 | | 0.4408 | 0.6709 | -0.889 | 1.771 | 0.51 |
| **% (Neo)Adjuvant CT** | 41 | | 0.2568 | 0.6288 | 1.015 | 1.529 | 0.69 |
| **% Primary CCRT** | 39 | | 1.1378 | 0.4783 | 0.169 | 2.107 | 0.02* |
| **% Laryngectomy** | 18 | | -1.2789 | 0.4370 | -2.205 | -0.353 | <0.01* |
| **% Pain** | 8 | | -1.7750 | 0.9069 | -3.994 | 0.444 | 0.10 |
| **% Dysphagia** | 10 | | 0.4259 | 0.8105 | -1.443 | 2.295 | 0.61 |
| **Anxiety Disorders** | | | | | | | |
| **NOS Scale** | 17 | | -0.0587 | 0.4191 | -0.952 | 0.835 | 0.89 |
| **Publication year** | 17 | | -0.0423 | 0.0372 | -0.122 | 0.037 | 0.27 |
| **Mean age** | 14 | | 0.0916 | 0.0674 | -0.055 | 0.238 | 0.20 |
| **% Female** | 16 | | 4.882 | 2.8135 | -1.146 | 10.923 | 0.10 |
| **% Alcohol use** | 8 | | 2.6806 | 1.7666 | -1.642 | 7.003 | 0.18 |
| **% Tobacco use** | 9 | | -0.4921 | 1.0886 | -3.066 | 2.082 | 0.66 |
| **% Married** | 8 | | -2.0434 | 2.2698 | -7.597 | 3.511 | 0.40 |
| **% Larynx & Hypopharynx** | 13 | | 0.0749 | 0.7926 | -1.668 | 1.819 | 0.93 |
| **% Oropharynx** | 10 | | -0.6737 | 0.7669 | -2.442 | 1.095 | 0.41 |
| **% Oral cavity** | 13 | | 0.6065 | 0.7840 | -1.119 | 2.332 | 0.45 |
| **% Initial stage (I, II)** | 12 | | -0.4141 | 1.1067 | -2.880 | 2.052 | 0.72 |
| **% Advanced stage (III, IV)** | 14 | | -0.4913 | 1.123 | -2.939 | 1.957 | 0.67 |
| **% Surgery (total)** | 10 | | 2.1645 | 1.4597 | -1.202 | 5.531 | 0.18 |
| **% RT (total)** | 11 | | 0.8557 | 2.0626 | -3.820 | 5.512 | 0.69 |
| **Distress (Symptoms)** | | | | | | | |
| **NOS Scale** | 39 | | 0.1240 | 0.0947 | -0.068 | 0.316 | 0.20 |
| **Publication year** | 39 | | 0.0270 | 0.0190 | -0.011 | 0.065 | 0.16 |
| **Mean age** | 29 | | -0.0460 | 0.0229 | -0.093 | 0.001 | 0.05 |
| **% Female** | 35 | | -2.5190 | 0.8117 | -4.170 | -0.869 | <0.01* |
| **% Alcohol use** | 9 | | -0.6652 | 0.7826 | -1.186 | 2.516 | 0.42 |
| **% Tobacco use** | 10 | | 1.6589 | 0.5934 | 0.290 | 3.027 | 0.02* |
| **% Low education** | 12 | | 0.3156 | 0.8909 | -1.669 | 2.301 | 0.73 |
| **% High education** | 11 | | -3.7028 | 1.1406 | -6.894 | -0.512 | 0.02* |
| **% Employed** | 11 | | 1.5706 | 0.7426 | -0.109 | 3.251 | 0.06 |
| **% Married** | 18 | | -1.4128 | 1.1586 | -3.869 | 1.043 | 0.24 |
| **% Larynx & Hypopharynx** | 29 | | -0.8592 | 0.6004 | -2.091 | 0.373 | 0.16 |
| **% Oropharynx** | 17 | | 0.4382 | 1.1861 | -2.089 | 2.966 | 0.72 |
| **% Oral cavity** | 27 | | -0.4028 | 0.5059 | -1.445 | 0.639 | 0.43 |
| **% Initial stage (I, II)** | 25 | | -1.8231 | 0.5131 | -2.884 | -0.762 | <0.01* |
| **% Advanced stage (III, IV)** | 25 | | 1.2827 | 0.5349 | 0.1766 | 2.389 | 0.03* |
| **% Surgery (total)** | 23 | | -0.3971 | 0.4939 | -1.424 | 0.630 | 0.43 |
| **% Only surgery** | 19 | | -1.2492 | 0.8323 | -3.005 | 0.507 | 0.15 |
| **% Multimodal surgery** | 19 | | -0.1486 | 0.7010 | -1.628 | 1.330 | 0.83 |
| **% RT (total)** | 25 | | -0.082 | 0.6198 | -1.290 | 1.274 | 0.99 |
| **% Only RT** | 16 | | -1.4212 | 0.6606 | -2.838 | -0.004 | 0.04* |
| **% (Neo)Adjuvant CT** | 11 | | 0.9486 | 0.9027 | -1.094 | 2.991 | 0.32 |
| **% Primary CCRT** | 18 | | 3.1408 | 0.5829 | 1.905 | 4.376 | <0.01 |

**Supplementary Table S7: Subgroup analyses for the studied outcomes**

|  | **No. Studies** | **Sample size** | **Proportion** | **95% CI** | **Heterogeneity** | |
| --- | --- | --- | --- | --- | --- | --- |
|  |  |  |  |  | **I^2^ (%)** | ***p*** |
| **Depressive Symptoms** | | | | | | |
| **Continent** | Test for between groups difference: Q = 3.78; p = 0.58 | | | | | |
| **Measurement point** | Test for between groups difference: Q = 4.47; p = 0.72 | | | | | |
| **Depressive Disorders** | | | | | | |
| **Continent** | Test for between groups difference: Q = 0.43; p = 0.81 | | | | | |
| **Measurement point** | Test for between groups difference: Q = 1.28; p = 0.86 | | | | | |
| **Anxiety Symptoms** | | | | | | |
| **Continent** | Test for between groups difference: Q = 3.68; p = 0.45 | | | | | |
| **Measurement point** | Test for between groups difference: Q = 3.16; p = 0.53 | | | | | |
| **Anxiety Disorders** | | | | | | |
| **Continent** | Test for between groups difference: Q = 4.18; p = 0.24 | | | | | |
| **Measurement point** | Test for between groups difference: Q = 13.98; p < 0.01* | | | | | |
| Pre-Treatment | 3 | 2782 | 0.103 | 0.054 - 0.190 | 0.00 | 0.82 |
| During treatment | 4 | 1278 | 0.038 | 0.009 - 0.153 | 90.0 | 0.00 |
| 1st year after treatment | 2 | 316 | 0.069 | 0.000 - 0.999 | 89.1 | 0.00 |
| >1 year after treatment | 2 | 840 | 0.178 | 0.011 - 0.812 | 55.1 | 0.04 |
| **Distress Symptoms** | | | | | | |
| **Continent** | Test for between groups difference: Q = 16.07; p < 0.01* | | | | | |
| Europe | 23 | 2925 | 0.304 | 0.254 - 0.359 | 83.3 | 0.01 |
| North America | 5 | 475 | 0.378 | 0.174 - 0.636 | 90.0 | 0.00 |
| Asia | 8 | 1579 | 0.408 | 0.323 - 0.499 | 89.6 | 0.00 |
| South America | 1 | 50 | 0.580 | 0.441 - 0.708 | N.a. | N.a. |
| Oceania | 2 | 84 | 0.377 | 0.001 - 0.998 | 88.9 | 0.00 |
| **Measurement point** | Test for between groups difference: Q = 16.90; p < 0.01* | | | | | |
| Pre-Treatment | 8 | 1952 | 0.413 | 0.342 - 0.487 | 89.2 | 0.00 |
| During treatment | 5 | 555 | 0.412 | 0.215 - 0.647 | 94.3 | 0.00 |
| 1st year after treatment | 7 | 539 | 0.387 | 0.251 - 0.544 | 79.8 | 0.01 |
| >1 year after treatment | 13 | 1562 | 0.275 | 0.234 - 0.319 | 61.4 | 0.03 |

**Supplementary Figure 2: Forest plots for the studied outcomes**


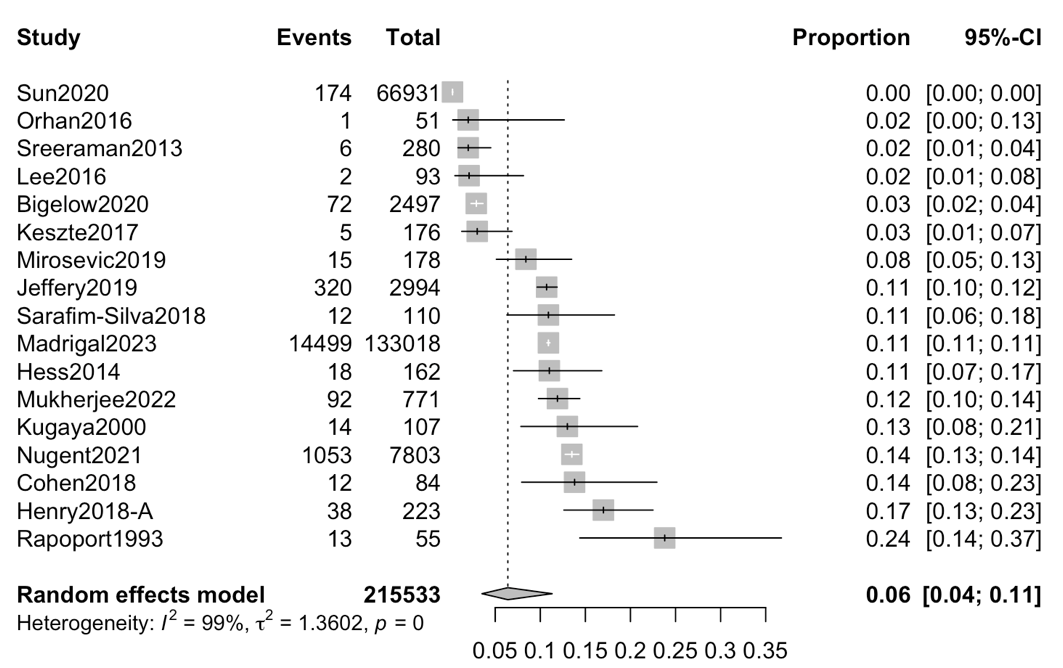

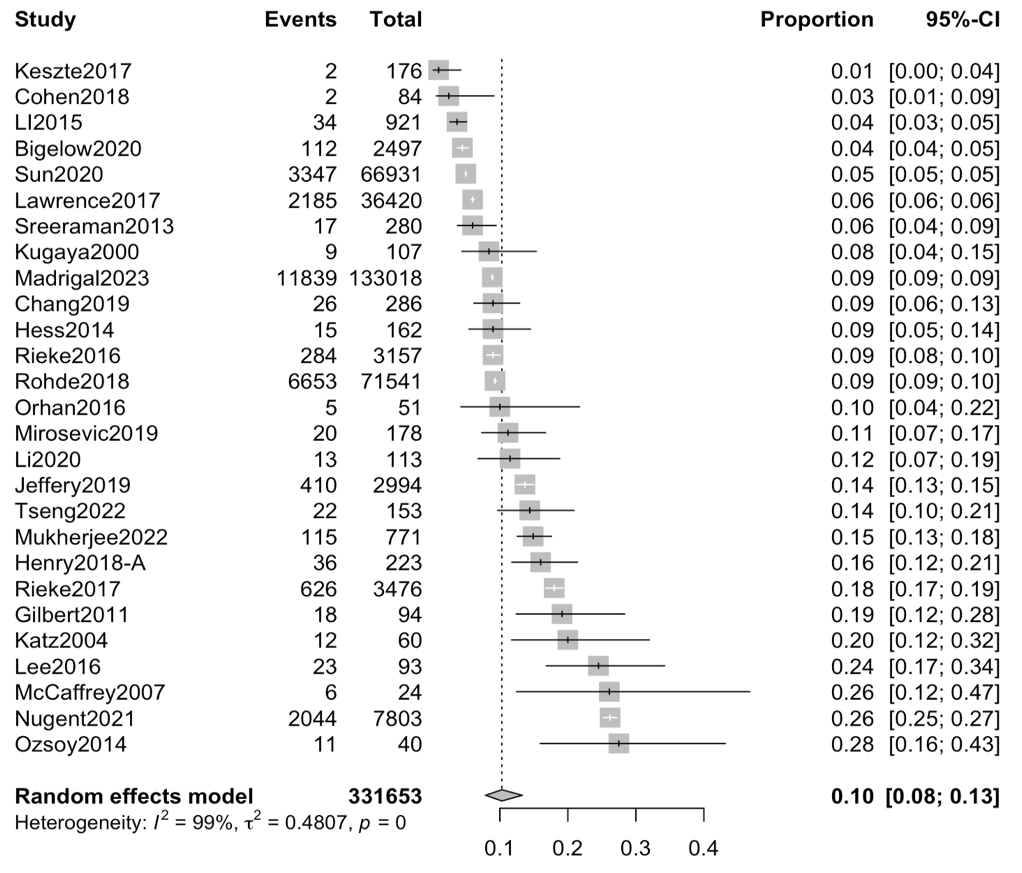


**Supplementary Figure 2-B**. Forest plot for prevalence of anxiety disorders.

**Supplementary Figure 2-A**. Forest plot for prevalence of depressive disorders.


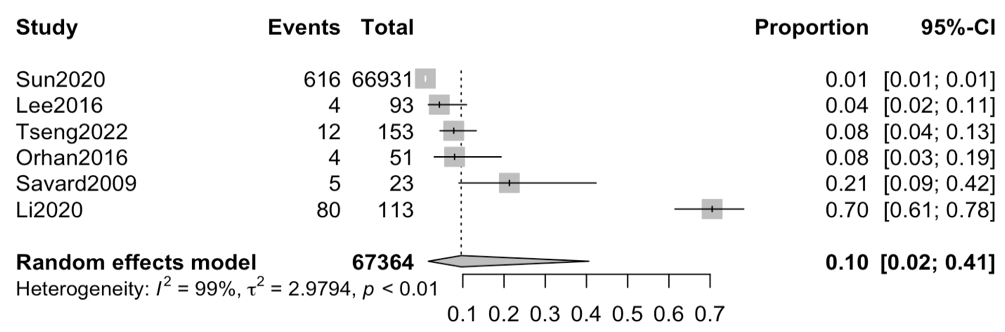


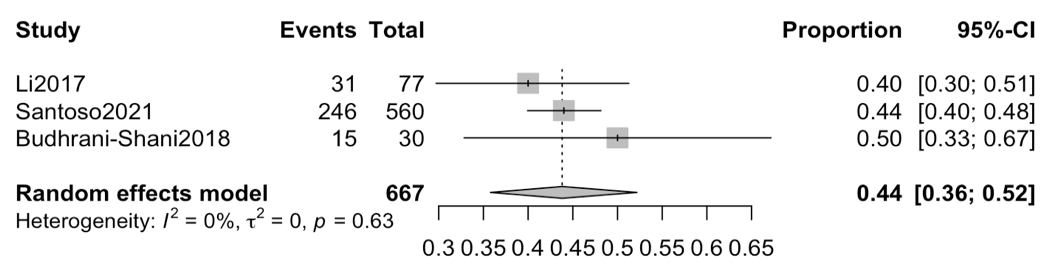


**Supplementary Figure 2-D**. Forest plot for prevalence of insomnia disorder.

**Supplementary Figure 2-C**. Forest plot for prevalence of insomnia related symptoms.


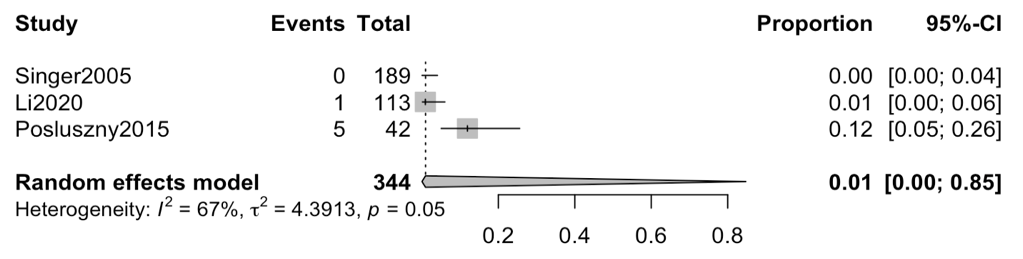

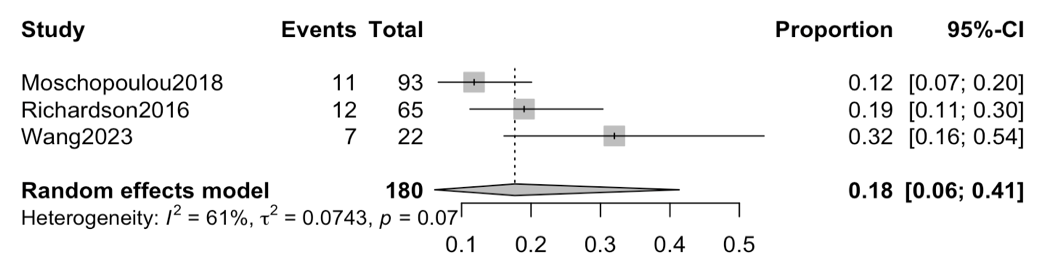

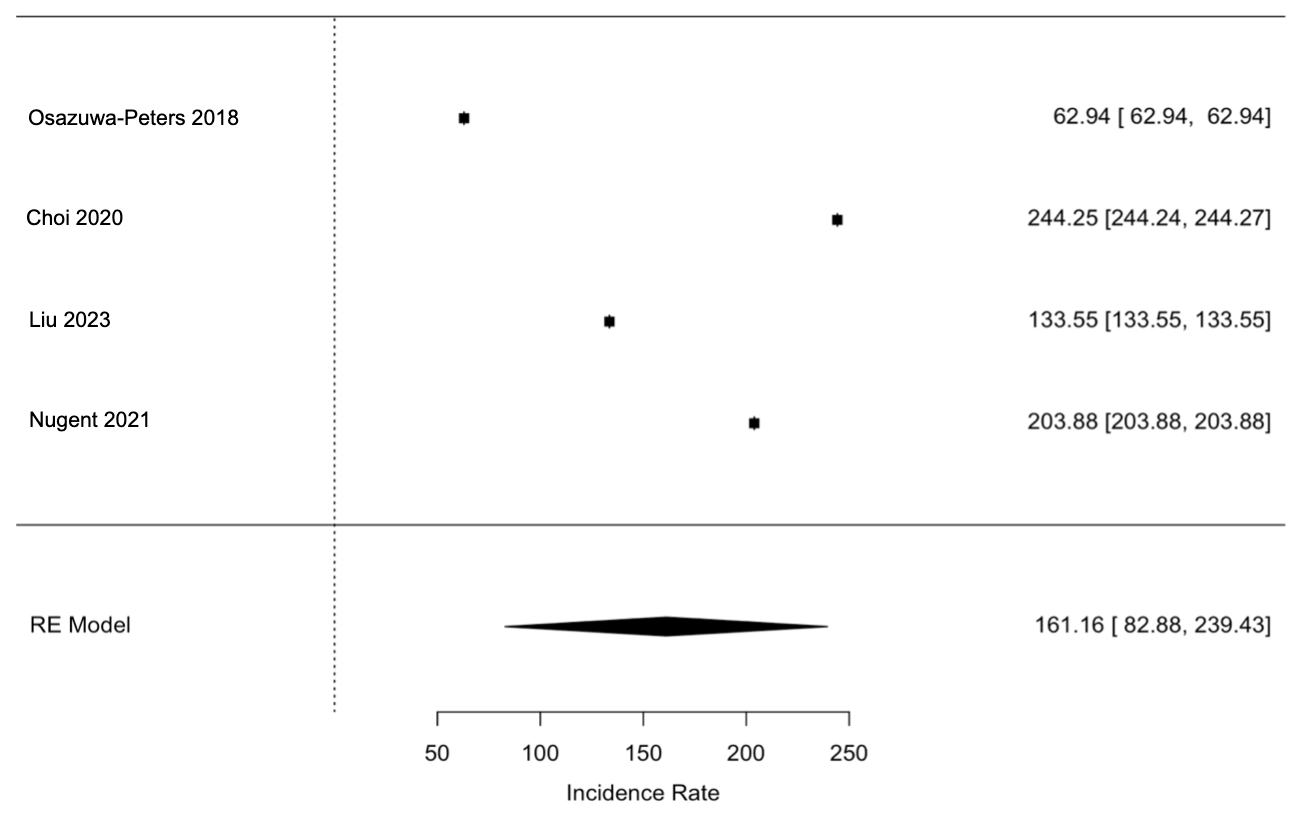


**Supplementary Figure 2-E**. Forest plot for prevalence of post-traumatic stress symptoms.

**Supplementary Figure 2-G**. Forest plot for incidence of suicide.

**Supplementary Figure 2-F**. Forest plot for prevalence of post-traumatic stress disorder.


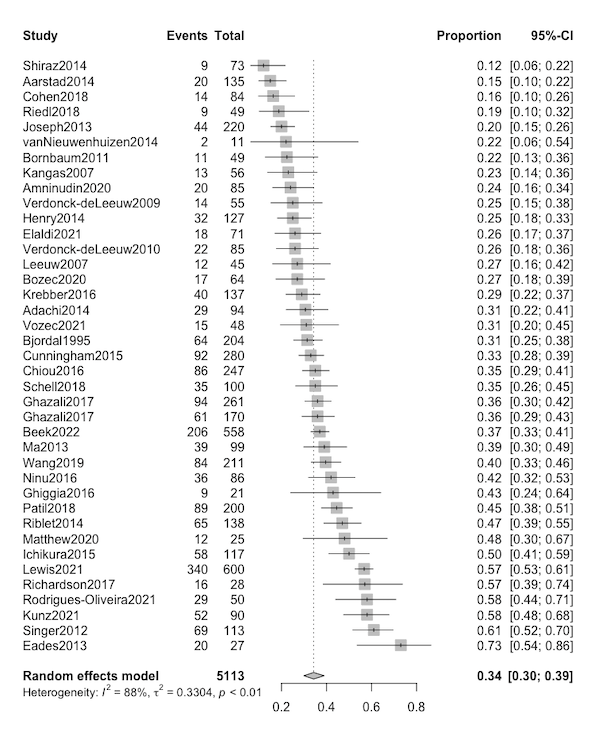


**Supplementary Figure 2-H.** Forest plot for clinically significant distress. CI Confidence Interval.

**REFERENCES**

1. Bastos DB, Sarafim-Silva BAM, Sundefeld MLMM, et al. Circulating catecholamines are associated with biobehavioral factors and anxiety symptoms in head and neck cancer patients*. PloS one*. 2018;13(8):e0202515. doi: 10.1371/journal.pone.0202515.

2. Menezes ASdS, Sanches GLG, Gomes ESB, et al. The combination of traditional and auricular acupuncture to prevent xerostomia and anxiety in irradiated patients with HNSCC: a preventive, parallel, single-blind, 2-arm controlled study*. Oral surgery, oral medicine, oral pathology and oral radiology*. 2021;131(6):675-683. doi: 10.1016/j.oooo.2021.02.016.

3. BIRKHAUG EJ, AARSTAD HJ, AARSTAD AKH, OLOFSSON J. Relation between mood, social support and the quality of life in patients with laryngectomies*. European archives of oto-rhino-laryngology*. 2002;259(4):197-204. doi: 10.1107/s00405-001-0444-8.

4. Byrne A, Walsh M, Farrelly M, O'Driscoll K. Depression following laryngectomy. A pilot study*. British journal of psychiatry*. 1993;163(2):173-176. doi: 10.1192/bjp.163.2.173.

5. Chan JYK, Lua LL, Starmer HH, Sun DQ, Rosenblatt ES, Gourin CG. The relationship between depressive symptoms and initial quality of life and function in head and neck cancer*. The Laryngoscope*. 2011;121(6):1212-1218. doi: 10.1002/lary.21788.

6. Chang MY, Rogers SN, Lowe D, et al. The Korean version of the University of Washington Quality of Life Questionnaire for Patients with Head and Neck Cancer, and its use in an initial validation study of 56 patients*. International journal of oral and maxillofacial surgery*. 2012;41(10):1201-1205. doi: 10.1016/j.ijom.2012.05.016.

7. Chawla S, Mohanti BK, Rakshak M, Saxena S, Rath GK, Bahadur S. Temporal Assessment of Quality of Life of Head and Neck Cancer Patients Receiving Radical Radiotherapy*. Quality of life research*. 1999;8(1/2):73-78. doi: 10.1023/A:1026476928283.

8. Chiou W, Lee M, Ho H, et al. Prognosticators and the relationship of depression and quality of life in head and neck cancer*. Indian journal of cancer*. 2013;50(1):14-20. doi: 10.4103/0019-509X.112279.

9. D'Antonio LL, Long SA, Zimmerman GJ, Peterman AH, Petti GH, Chonkich GD. Relationship Between Quality of Life and Depression in Patients With Head and Neck Cancer*. The Laryngoscope*. 1998;108(6):806-811. doi: 10.1097/00005537-199806000-00006.

10. El-Deiry M, Funk GF, Nalwa S, et al. Long-term Quality of Life for Surgical and Nonsurgical Treatment of Head and Neck Cancer*. Archives of otolaryngology, head & neck surgery*. 2005;131(10):879-885. doi: 10.1001/archotol.131.10.879.

11. Givens DJ, Karnell LH, Gupta AK, et al. Adverse Events Associated With Concurrent Chemoradiation Therapy in Patients With Head and Neck Cancer*. Archives of otolaryngology--head & neck surgery*. 2009;135(12):1209-1217. doi: 10.1001/archoto.2009.174.

12. Haisfield-Wolfe ME, McGuire DB, Soeken K, Geiger-Brown J, De Forge B, Suntharalingam M. Prevalence and correlates of symptoms and uncertainty in illness among head and neck cancer patients receiving definitive radiation with or without chemotherapy*. Support Care Cancer*. 2012;20(8):1885-1893. doi: 10.1007/s00520-011-1291-9.

13. Howren MB, Christensen AJ, Karnell LH, Funk GF. Health-Related Quality of Life in Head and Neck Cancer Survivors*. Health psychology*. 2010;29(1):65-71. doi: 10.1037/a0017788.

14. Howren MB, Seaman A, Christensen AJ, Pagedar NA. Association of depressive symptomatology with problem alcohol use in rural head and neck cancer patients at diagnosis*. Journal of psychosocial oncology*. 2022;40(6):868-880. doi: 10.1080/07347332.2021.1971816.

15. Kim S, Roh J, Lee S, et al. Pretreatment depression as a prognostic indicator of survival and nutritional status in patients with head and neck cancer*. Cancer*. 2016;122(1):131-140. doi: 10.1002/cncr.29693.

16. Pytel A, Zielińska A, Staś J, Chabowski M. Quality of Life, Psychological Distress, and Nutritional Status of Polish Patients with Head and Neck Cancer Treated with Radiotherapy*. Journal of clinical medicine*. 2023;12(2):659. doi: 10.3390/jcm12020659.

17. Terrell JE, Fisher SG, Wolf GT. Long-term Quality of Life After Treatment of Laryngeal Cancer*. Archives of otolaryngology--head & neck surgery*. 1998;124(9):964-971. doi: 10.1001/archotol.124.9.964.

18. Sarafim‐Silva BAM, Duarte GD, Sundefeld MLMM, Biasoli ÉR, Miyahara GI, Bernabé DG. Childhood trauma is predictive for clinical staging, alcohol consumption, and emotional symptoms in patients with head and neck cancer*. Cancer*. 2018;124(18):3684-3692. doi: 10.1002/cncr.31597.

19. Karnell LH, Funk GF, Christensen AJ, Rosenthal EL, Magnuson JS. Persistent posttreatment depressive symptoms in patients with head and neck cancer*. Head & neck*. 2006;28(5):453-461. doi: 10.1002/hed.20370.

20. Bakhshaie J, Bonnen M, Asper J, Sandulache V, Badr H. Emotional disclosure and cognitive processing in couples coping with head and neck cancer*. J Behav Med*. 2020;43(3):411-425. doi: 10.1007/s10865-019-00094-5.

21. Astrup G, Rustøen T, Miaskowski C, Paul S, Bjordal K. A Longitudinal Study of Depressive Symptoms in Patients With Head and Neck Cancer Undergoing Radiotherapy*. Cancer nursing*. 2015;38(6):436-446. doi: 10.1097/NCC.0000000000000225.

22. VAN DER SCHROEFF MP, DERKS W, HORDIJK GJ, DE LEEUW RJ. The effect of age on survival and quality of life in elderly head and neck cancer patients: a long-term prospective study*. European archives of oto-rhino-laryngology*. 2007;264(4):415-422. doi: 10.1007/s00405-006-0203-y.

23. Nightingale CL, Lagorio L, Carnaby G. A Prospective Pilot Study of Psychosocial Functioning in Head and Neck Cancer Patient-Caregiver Dyads*. Journal of psychosocial oncology*. 2014;32(5):477-492. doi: 10.1080/07347332.2014.936649.

24. Chhabria KS, Carnaby GD. Psychometric validation of the Center for Epidemiological Studies Depression Scale in Head and Neck Cancer patients*. Oral oncology*. 2017;75:158-162. doi: 10.1016/j.oraloncology.2017.11.010.

25. Irish J, Sandhu N, Simpson C, et al. Quality of life in patients with maxillectomy prostheses*. Head & neck*. 2009;31(6):813-821. doi: 10.1002/hed.21042.

26. Katz MR, Irish JC, Devins GM, Rodin GM, Gullane PJ. Psychosocial adjustment in head and neck cancer: The impact of disfigurement, gender and social support*. Head & neck*. 2003;25(2):103-112. doi: 10.1002/hed.10174.

27. de Leeuw JRJ, de Graeff A, Ros WJG, Blijham GH, Hordijk G, Winnubst JAM. Prediction of depression 6 months to 3 years after treatment of head and neck cancer*. Head & neck*. 2001;23(10):892-898. doi: 10.1002/hed.1129.

28. Monga U, Tan G, Ostermann HJ, Monga TN. Sexuality in head and neck cancer patients*. Archives of physical medicine and rehabilitation*. 1997;78(3):298-304. doi: 10.1016/S0003-9993(97)90038-1.

29. Morse DE, Psoter WJ, Baek LS, et al. Smoking and drinking in relation to depressive symptoms among persons with oral cancer or oral epithelial dysplasia*. Head & neck*. 2010;32(5):578-587. doi: 10.1002/hed.21227.

30. Qualliotine JR, Califano JA, Li RJ, et al. Human papillomavirus tumour status is not associated with a positive depression screen for patients with oropharyngeal cancer*. Journal of laryngology and otology*. 2017;131(9):760-767. doi: 10.1017/S0022215117001098.

31. Ren JL, Rojo RD, Perez JVD, Yeung SJ, Hanna EY, Reyes-Gibby CC. Variations in pain prevalence, severity, and analgesic use by duration of survivorship: a cross-sectional study of 505 post-treatment head and neck cancer survivors*. BMC cancer*. 2021;21(1):1304. doi: 10.1186/s12885-021-09024-8.

32. Rhoten BA, Murphy BA, Dietrich MS, Ridner SH. Depressive symptoms, social anxiety, and perceived neck function in patients with head and neck cancer*. Head & neck*. 2018;40(7):1443-1452. doi: 10.1002/hed.25129.

33. Rogers LQ, Courneya KS, Robbins KT, et al. Factors associated with fatigue, sleep, and cognitive function among patients with head and neck cancer*. Head & neck*. 2008;30(10):1310-1317. doi: 10.1002/hed.20873.

34. Speksnijder CM, Lankhorst PJM, de Bree R, de Haan AFJ, Koole R, Merkx MAW. Depression and related factors after oral oncological treatment: a 5-year prospective cohort study*. Support Care Cancer*. 2021;29(6):2907-2916. doi: 10.1007/s00520-020-05795-1.

35. Meulen IC, May AM, Ros WJG, et al. One‐Year Effect of a Nurse‐Led Psychosocial Intervention on Depressive Symptoms in Patients With Head and Neck Cancer: A Randomized Controlled Trial*. The oncologist (Dayton, Ohio)*. 2013;18(3):336-344. doi: 10.1634/theoncologist.2012-0299.

36. van Wilgen CP, Dijkstra PU, van der Laan, B. F. A. M., Plukker JT, Roodenburg JLN. Shoulder and neck morbidity in quality of life after surgery for head and neck cancer*. Head & neck*. 2004;26(10):839-844. doi: 10.1002/hed.20052.

37. Liu F, Huang J, Lin C, Kuo T. Suicide risk after head and neck cancer diagnosis in Taiwan: A retrospective cohort study*. Journal of affective disorders*. 2023;320:610-615. doi: 10.1016/j.jad.2022.09.151.

38. Henry M, Rosberger Z, Bertrand L, et al. Prevalence and Risk Factors of Suicidal Ideation among Patients with Head and Neck Cancer: Longitudinal Study*. Otolaryngology-head and neck surgery*. 2018;159(5):843-852. doi: 10.1177/0194599818776873.

39. Gilbert J, Haman KL, Dietrich MS, Blakely RD, Shelton RC, Murphy BA. Depression in patients with head and neck cancer and a functional genetic polymorphism of the serotonin transporter gene*. Head & neck*. 2012;34(3):359-364. doi: 10.1002/hed.21744.

40. McCaffrey JC, Weitzner M, Kamboukas D, Haselhuhn G, LaMonde L, Booth-Jones M. Alcoholism, depression, and abnormal cognition in head and neck cancer: A pilot study*. Otolaryngology-head and neck surgery*. 2007;136(1):92-97. doi: 10.1016/j.otohns.2006.06.1275.

41. Ozsoy S, M.D, Besirli A, M.D, Unal D, M.D, Abdulrezzak U, M.D, Orhan O, M.D. The association between depression, weight loss and leptin/ghrelin levels in male patients with head and neck cancer undergoing radiotherapy*. General hospital psychiatry*. 2015;37(1):31-35. doi: 10.1016/j.genhosppsych.2014.09.002.

42. Hess CB, Rash DL, Daly ME, et al. Competing Causes of Death and Medical Comorbidities Among Patients With Human Papillomavirus–Positive vs Human Papillomavirus–Negative Oropharyngeal Carcinoma and Impact on Adherence to Radiotherapy*. JAMA otolaryngology-- head & neck surgery*. 2014;140(4):312-316. doi: 10.1001/jamaoto.2013.6732.

43. Keszte J, Danker H, Dietz A, et al. Course of psychiatric comorbidity and utilization of mental health care after laryngeal cancer: a prospective cohort study*. Eur Arch Otorhinolaryngol*. 2017;274(3):1591-1599. doi: 10.1007/s00405-016-4340-7.

44. Kugaya A, Akechi T, Okuyama T, et al. Prevalence, predictive factors, and screening for psychologic distress in patients with newly diagnosed head and neck cancer*. Cancer*. 2000;88(12):2817-2823. doi: 10.1002/1097-0142(20000615)88:123.0.CO;2-N.

45. Sreeraman R, Vijayakumar S, Chen AM. Correlation of radiation treatment interruptions with psychiatric disease and performance status in head and neck cancer patients*. Support Care Cancer*. 2013;21(12):3301-3306. doi: 10.1007/s00520-013-1907-3.

46. Lee Y, Wu Y, Chien C, Fang F, Hung C. Use of the Hospital Anxiety and Depression Scale and the Taiwanese Depression Questionnaire for screening depression in head and neck cancer patients in Taiwan*. Neuropsychiatric Disease and Treatment*. 2016;12:2649-2657. doi: 10.2147/NDT.S112069.

47. Unal D, Orhan O, Ozsoy S, Besirli A, Eroglu C, Kaplan B. Effect of radiotherapy on psychiatric disorder in patients with head and neck cancer*. Indian journal of cancer*. 2016;53(1):162-165. doi: 10.4103/0019-509X.180816.

48. Lee Y, Hung C, Chien C, et al. Comparison of prevalence and associated factors of depressive disorder between patients with head and neck cancer and those with lung cancer at a tertiary hospital in Taiwan: a cross-sectional study*. BMJ open*. 2020;10(6):e037918. doi: 10.1136/bmjopen-2020-037918.

49. Aminnudin AN, Doss JG, Ismail SM, et al. Can post-treatment oral cancer patients’ concerns reflect their cancer characteristics, HRQoL, psychological distress level and satisfaction with consultation?*. Ecancermedicalscience*. 2020;14:1118. doi: 10.3332/ecancer.2020.1118.

50. Chiou Y, Chiu N, Wang L, et al. Prevalence and related factors of psychological distress among cancer inpatients using routine Distress Thermometer and Chinese Health Questionnaire screening*. Neuropsychiatric Disease and Treatment*. 2016;12:2765-2773. doi: 10.2147/NDT.S118667.

51. Wells M, Cunningham M, Lang H, et al. Distress, concerns and unmet needs in survivors of head and neck cancer: a cross-sectional survey*. European journal of cancer care*. 2015;24(5):748-760. doi: 10.1111/ecc.12370.

52. Eades M, Murphy J, Carney S, et al. Effect of an interdisciplinary rehabilitation program on quality of life in patients with head and neck cancer: Review of clinical experience*. Head & neck*. 2013;35(3):343-349. doi: 10.1002/hed.22972.

53. Ghazali N, Roe B, Lowe D, et al. Using the patients concerns inventory for distress screening in post-treatment head and neck cancer survivors*. Journal of cranio-maxillo-facial surgery*. 2017;45(10):1743-1748. doi: 10.1016/j.jcms.2017.07.009.

54. Ghazali N, Roe B, Lowe D, et al. Screening for distress using the distress thermometer and the University of Washington Quality of Life in post-treatment head and neck cancer survivors*. Eur Arch Otorhinolaryngol*. 2017;274(5):2253-2260. doi: 10.1007/s00405-017-4474-2.

55. Kunz V, Wichmann G, Lehmann-Laue A, Mehnert-Theuerkauf A, Dietz A, Wiegand S. Screening for distress, related problems and perceived need for psycho-oncological support in head and neck squamous cell carcinoma (HNSCC) patients: a retrospective cohort study*. BMC cancer*. 2021;21(1):478. doi: 10.1186/s12885-021-08236-2.

56. Lewis S, Pandey S, Salins N, et al. Distress Screening in Head and Neck Cancer Patients Planned for Cancer‐Directed Radiotherapy*. The Laryngoscope*. 2021;131(9):2023-2029. doi: 10.1002/lary.29491.

57. Ma L, Poulin P, Feldstain A, Chasen MR. The Association between Malnutrition and Psychological Distress in Patients with Advanced Head-and-Neck Cancer*. Current oncology (Toronto)*. 2013;20(6):554. doi: 10.3747/co.20.1651.

58. Mathew B, Vidhubala E, Krishnamurthy A, Sundaramoorthy C. Can Cancer Diagnosis Help in Quitting Tobacco? Barriers and Enablers to Tobacco Cessation Among Head and Neck Cancer Patients from a Tertiary Cancer Center in South india*. Indian journal of psychological medicine*. 2020;42(4):346-352. doi: 10.1177/0253717620930317.

59. Ninu MB, Miccinesi G, Bulli F, et al. Psychological Distress and Health-Related Quality of Life among Head and Neck Cancer Patients during the First Year after Treatment*. Tumori*. 2016;102(1):96-102. doi: 10.5301/tj.5000448.

60. Patil V, Noronha V, Joshi A, et al. Distress Management in Patients With Head and Neck Cancer Before Start of Palliative Chemotherapy: A Practical Approach*. Journal of Global Oncology*. 2018;4(4):1-10. doi: 10.1200/JGO.17.00044.

61. Riblet N, Skalla K, McClure A, Homa K, Luciano A, Davis TH. Addressing Distress in Patients With Head and Neck Cancers: A Mental Health Quality Improvement Project*. Journal of the National Comprehensive Cancer Network*. 2014;12(7):1005-1013. doi: 10.6004/jnccn.2014.0097.

62. Schell J, Petermann-Meyer A, Kloss-Brandstätter A, et al. Distress thermometer for preoperative screening of patients with oral squamous cell carcinoma*. Journal of cranio-maxillo-facial surgery*. 2018;46(7):1111-1116. doi: 10.1016/j.jcms.2018.04.022.

63. Bultz BD, Waller A, Cullum J, et al. Implementing Routine Screening for Distress, the Sixth Vital Sign, for Patients With Head and Neck and Neurologic Cancers*. Journal of the National Comprehensive Cancer Network*. 2013;11(10):1249-1261. doi: 10.6004/jnccn.2013.0147.

64. Duffy SA, Terrell JE, Valenstein M, Ronis DL, Copeland LA, Connors M. Effect of smoking, alcohol, and depression on the quality of life of head and neck cancer patients*. General hospital psychiatry*. 2002;24(3):140-147. doi: 10.1016/S0163-8343(02)00180-9.

65. Duffy, Sonia A., Ph.D., R.N, Ronis DL, Ph.D, Valenstein M, M.D, et al. Depressive Symptoms, Smoking, Drinking, and Quality of Life Among Head and Neck Cancer Patients*. Psychosomatics (Washington, D.C.)*. 2007;48(2):142-148. doi: 10.1176/appi.psy.48.2.142.

66. Duffy SA, Khan MJ, Ronis DL, et al. Health behaviors of head and neck cancer patients the first year after diagnosis*. Head & neck*. 2008;30(1):93-102. doi: 10.1002/hed.20665.

67. Lambert M, Terrell J, Copeland L, Ronis D, Duffy S. Cigarettes, alcohol, and depression: Characterizing head and neck cancer survivors in two systems of care*. Nicotine & tobacco research*. 2005;7(2):233-241. doi: 10.1080/14622200500055418.

68. Shuman AG, Duffy SA, Ronis DL, et al. Predictors of poor sleep quality among head and neck cancer patients*. The Laryngoscope*. 2010;120(6):1166-1172. doi: 10.1002/lary.20924.

69. Shuman AG, Terrell JE, Light E, et al. Predictors of Pain Among Patients With Head and Neck Cancer*. Archives of otolaryngology--head & neck surgery*. 2012;138(12):1147-1154. doi: 10.1001/jamaoto.2013.853.

70. Thomas CM, Sklar MC, Su J, et al. Evaluation of Older Age and Frailty as Factors Associated With Depression and Postoperative Decision Regret in Patients Undergoing Major Head and Neck Surgery*. Archives of otolaryngology--head & neck surgery*. 2019;145(12):1170-1178. doi: 10.1001/jamaoto.2019.3020.

71. BJORDAL K, KAASA S. Psychological distress in head and neck cancer patients 7-11 years after curative treatment*. British Journal of Cancer*. 1995;71(3):592-597. doi: 10.1038/bjc.1995.115.

72. Aarstad HJ, Osthus AA, Olofsson J, Aarstad AKH. Level of distress predicts subsequent survival in successfully treated head and neck cancer patients: a prospective cohort study*. Acta oto-laryngologica*. 2014;134(2):211-219. doi: 10.3109/00016489.2013.841989.

73. Richardson AE, Tennant G, Morton RP, Broadbent E. A Self-Regulatory Intervention for Patients with Head and Neck Cancer: Pilot Randomized Trial*. ann behav med*. 2017;51(5):629-641. doi: 10.1007/s12160-017-9885-1.

74. Adachi Y, Kimura H, Sato N, et al. Preoperative Level of Depression is a Predictor of Postoperative Levels of Depression in Patients with Head and Neck Cancer*. Japanese journal of clinical oncology*. 2014;44(4):311-317. doi: 10.1093/jjco/hyu002.

75. Elaldi R, Roussel L, Gal J, et al. Correlations between long-term quality of life and patient needs and concerns following head and neck cancer treatment and the impact of psychological distress. A multicentric cross-sectional study*. Eur Arch Otorhinolaryngol*. 2021;278(7):2437-2445. doi: 10.1007/s00405-020-06326-8.

76. Henry M, Habib L, Morrison M, et al. Head and neck cancer patients want us to support them psychologically in the posttreatment period: Survey results*. Palliative & supportive care*. 2014;12(6):481-493. doi: 10.1017/S1478951513000771.

77. Ichikura K, Yamashita A, Sugimoto T, Kishimoto S, Matsushima E. Persistence of psychological distress and correlated factors among patients with head and neck cancer*. Palliative & supportive care*. 2016;14(1):42-51. doi: 10.1017/S1478951515000711.

78. Krebber AH, Jansen F, Cuijpers P, Leemans CR, Verdonck-de Leeuw IM. Screening for psychological distress in follow-up care to identify head and neck cancer patients with untreated distress*. Support Care Cancer*. 2016;24(6):2541-2548. doi: 10.1007/s00520-015-3053-6.

79. Verdonck-de Leeuw IM, Eerenstein SE, Van der Linden MH, Kuik DJ, de Bree R, Leemans CR. Distress in Spouses and Patients After Treatment for Head and Neck Cancer*. The Laryngoscope*. 2007;117(2):238-241. doi: 10.1097/01.mlg.0000250169.10241.58.

80. Singer S, Krauß O, Keszte J, et al. Predictors of emotional distress in patients with head and neck cancer*. Head & neck*. 2012;34(2):180-187. doi: 10.1002/hed.21702.

81. Wang Y, Lu W, Shen X. Assessment of preoperative psychologic distress in laryngeal cancer patients*. Acta oto-laryngologica*. 2019;139(2):184-186. doi: 10.1080/00016489.2018.1523555.

82. Cohen A, Ianovski LE, Frenkiel S, et al. Barriers to psychosocial oncology service utilization in patients newly diagnosed with head and neck cancer*. Psycho-oncology (Chichester, England)*. 2018;27(12):2786-2793. doi: 10.1002/pon.4889.

83. van Beek FE, Jansen F, Baatenburg de Jong RJ, et al. Psychological Problems among Head and Neck Cancer Patients in Relation to Utilization of Healthcare and Informal Care and Costs in the First Two Years after Diagnosis*. Current oncology (Toronto)*. 2022;29(5):3200-3214. doi: 10.3390/curroncol29050260.

84. Verdonck-de Leeuw IM, de Bree R, Keizer AL, et al. Computerized prospective screening for high levels of emotional distress in head and neck cancer patients and referral rate to psychosocial care*. Oral oncology*. 2009;45(10):e129-e133. doi: 10.1016/j.oraloncology.2009.01.012.

85. Allison PJ, Edgar L, Nicolau B, Archer J, Black M, Hier M. Results of a feasibility study for a psycho-educational intervention in head and neck cancer*. Psycho-oncology (Chichester, England)*. 2004;13(7):482-485. doi: 10.1002/pon.816.

86. Chang S, Lo C, Peng H, Chen C, Wu S, Chen S. Factors associated with continued smoking after treatment of oral cavity cancer: An age and survival time‐matched study*. Journal of advanced nursing*. 2018;74(4):926-934. doi: 10.1111/jan.13506.

87. Chen S, Huang B, Hung T, et al. Swallowing ability and its impact on dysphagia-specific health-related QOL in oral cavity cancer patients post-treatment*. European journal of oncology nursing : the official journal of European Oncology Nursing Society*. 2018;36:89-94. doi: 10.1016/j.ejon.2018.07.002.

88. Chen S, Huang B, Hung T, Lin C, Chang Y. Impact of a behavior change program and health education on social interactions in survivors of head and neck cancer: Randomized controlled trial*. Psycho-oncology (Chichester, England)*. 2019;28(2):293-300. doi: 10.1002/pon.4939.

89. Eadie T, Faust L, Bolt S, et al. Role of Psychosocial Factors on Communicative Participation among Survivors of Head and Neck Cancer*. Otolaryngology-head and neck surgery*. 2018;159(2):266-273. doi: 10.1177/0194599818765718.

90. HAMMERLID E, BJORDAL K, AHLNER-ELMQVIST M, et al. Prospective, longitudinal quality-of-life study of patients with head and neck cancer: A feasibility study including the EORTC QLQ-C30*. Otolaryngology-head and neck surgery*. 1997;116(6):666-673. doi: 10.1016/S0194-5998(97)70246-8.

91. Hartl DM, Dauchy S, Escande C, Bretagne E, Janot F, Kolb F. Quality of life after free-flap tongue reconstruction*. Journal of laryngology and otology*. 2009;123(5):550-554. doi: 10.1017/S0022215108003629.

92. Lee, Li-Yun, RN, MS, PhD, Chen, Shu-Ching, RN, PhD, Chen, Wen-Cheng, MD, BS, Huang, Bing-Shen, MD, PhD, Lin, Chien-Yu, MD, PhD. Postradiation trismus and its impact on quality of life in patients with head and neck cancer*. Oral surgery, oral medicine, oral pathology and oral radiology*. 2015;119(2):187-195. doi: 10.1016/j.oooo.2014.10.003.

93. Lee-Preston V, Steen IN, Dear A, et al. Optimizing the assessment of quality of life after laryngeal cancer treatment*. Journal of laryngology and otology*. 2004;118(6):432-438. doi: 10.1258/002221504323219554.

94. Patterson JM, Lu L, Watson L, et al. Associations between markers of social functioning and depression and quality of life in survivors of head and neck cancer: Findings from the Head and Neck Cancer 5000 study*. Psycho-oncology (Chichester, England)*. 2022;31(3):478-485. doi: 10.1002/pon.5830.

95. Wang C, Chen J, Su L, et al. The psychological status in patients with nasopharyngeal carcinoma during radiotherapy*. Eur Arch Otorhinolaryngol*. 2022;279(2):1035-1042. doi: 10.1007/s00405-021-06892-5.

96. Chen S, Lai Y, Liao C, et al. Supportive care needs in newly diagnosed oral cavity cancer patients receiving radiation therapy*. Psycho-oncology (Chichester, England)*. 2013;22(6):1220-1228. doi: 10.1002/pon.3126.

97. Hassanein KAM, Musgrove BT, Bradbury E. Psychological outcome of patients following treatment of oral cancer and its relation with functional status and coping mechanisms*. Journal of cranio-maxillo-facial surgery*. 2005;33(6):404-409. doi: 10.1016/j.jcms.2005.05.005.

98. Singer S, Herrmann E, Welzel C, Klemm E, Heim M, Schwarz R. Comorbid Mental Disorders in Laryngectomees*. Oncology research and treatment*. 2005;28(12):631-636. doi: 10.1159/000088978.

99. Aghajanzadeh S, Karlsson T, Tuomi L, Finizia C. The effect of jaw exercises on anxiety and depression in patients with head and neck cancer receiving radiotherapy: Prospective 2‐year follow‐up study*. Head & neck*. 2020;42(2):330-335. doi: 10.1002/hed.26012.

100. Airoldi M, Garzaro M, Raimondo L, et al. Functional and psychological evaluation after flap reconstruction plus radiotherapy in oral cancer*. Head & neck*. 2011;33(4):458-468. doi: 10.1002/hed.21471.

101. Almståhl A, Skoogh Andersson J, Alstad T, Fagerberg‐Mohlin B, Finizia C. Explorative study on quality of life in relation to salivary secretion rate in head and neck cancer patients treated with radiotherapy up to 2 years post treatment*. International journal of dental hygiene*. 2019;17(1):46-54. doi: 10.1111/idh.12363.

102. van Beek FE, Jansen F, Mak L, et al. The course of symptoms of anxiety and depression from time of diagnosis up to 2 years follow-up in head and neck cancer patients treated with primary (chemo)radiation*. Oral oncology*. 2020;102:104576. doi: 10.1016/j.oraloncology.2020.104576.

103. Berg M, Silander E, Bove M, Johansson L, Nyman J, Hammerlid E. Fatigue in Long‐Term Head and Neck Cancer Survivors From Diagnosis Until Five Years After Treatment*. The Laryngoscope*. 2023;133(9):2211-2221. doi: 10.1002/lary.30534.

104. Bernstein LJ, Pond GR, Gan HK, et al. Pretreatment neurocognitive function and self‐reported symptoms in patients with newly diagnosed head and neck cancer compared with noncancer cohort*. Head & neck*. 2018;40(9):2029-2042. doi: 10.1002/hed.25198.

105. Chaillou D, Mortuaire G, Deken-Delannoy V, Rysman B, Chevalier D, Mouawad F. Presence in head and neck cancer multidisciplinary team meeting: The patient's experience and satisfaction. European annals of otorhinolaryngology, head and neck diseases. 2019 Apr 1,;136(2):75.

106. Chen S, Liao C, Lin C, Chang JT, Lai Y. Distress and care needs in newly diagnosed oral cavity cancer patients receiving surgery*. Oral oncology*. 2009;45(9):815-820. doi: 10.1016/j.oraloncology.2009.01.001.

107. Chen Y, Lai Y, Lee Y, Tsai K, Chen M, Hsieh M. Impact of illness perception, mental adjustment, and sociodemographic characteristics on return to work in patients with head and neck cancer*. Support Care Cancer*. 2021;29(3):1519-1526. doi: 10.1007/s00520-020-05640-5.

108. D’Souza V, Blouin E, Zeitouni A, Muller K, Allison PJ. An investigation of the effect of tailored information on symptoms of anxiety and depression in Head and Neck cancer patients*. Oral oncology*. 2013;49(5):431-437. doi: 10.1016/j.oraloncology.2012.12.001.

109. Ehrsson YT, Fransson P, Einarsson S. Mapping Health-Related Quality of Life, Anxiety, and Depression in Patients with Head and Neck Cancer Diagnosed with Malnutrition Defined by GLIM*. Nutrients*. 2021;13(4):1167. doi: 10.3390/nu13041167.

110. Elani HW, Allison PJ. Coping and psychological distress among head and neck cancer patients*. Support Care Cancer*. 2011;19(11):1735-1741. doi: 10.1007/s00520-010-1013-8.

111. Finizia C, Hammerlid E, Westin T, Lindström J. Quality of life and voice in patients with laryngeal carcinoma: A posttreatment comparison of laryngectomy (salvage surgery) versus radiotherapy*. The Laryngoscope*. 1998;108(10):1566-1573. doi: 10.1097/00005537-199810000-00027.

112. Finizia C, Bergman B. Health-Related Quality of Life in Patients With Laryngeal Cancer: A Post-Treatment Comparison of Different Modes of Communication*. The Laryngoscope*. 2001;111(5):918-923. doi: 10.1097/00005537-200105000-00031.

113. Gosak M, Gradišar K, Rotovnik Kozjek N, Strojan P. Psychological distress and nutritional status in head and neck cancer patients: a pilot study*. Eur Arch Otorhinolaryngol*. 2020;277(4):1211-1217. doi: 10.1007/s00405-020-05798-y.

114. GRIFFITHS GO, PARMAR MKB, BAILEY AJ. Physical and psychological symptoms of quality of life in the CHART randomized trial in head and neck cancer: Short-term and long-term patient reported symptoms*. British Journal of Cancer*. 1999;81(7):1196-1205. doi: 10.1038/sj.bjc.6690829.

115. Hammerlid E, Mercke C, Sullivan M, Westin T. A prospective quality of life study of patients with oral or pharyngeal carcinoma treated with external beam irradiation with or without brachytherapy*. European journal of cancer. Part B, Oral oncology*. 1997;33(3):189-196. doi: 10.1016/S0964-1955(96)00069-3.

116. HAMMERLID E, AHINER-EIMQVIST M, BJORDAL K, et al. A prospective multicentre study in Sweden and Norway of mental distress and psychiatric morbidity in head and neck cancer patients*. British Journal of Cancer*. 1999;80(5-6):766-774. doi: 10.1038/sj.bjc.6690420.

117. Hammerlid E, Silander E, Hörnestam L, Sullivan M. Health-related quality of life three years after diagnosis of head and neck cancer-A longitudinal study*. Head & neck*. 2001;23(2):113-125. doi: 10.1002/1097-0347(200102)23:23.0.CO;2-W.

118. Horney DJ, Smith HE, McGurk M, et al. Associations between quality of life, coping styles, optimism, and anxiety and depression in pretreatment patients with head and neck cancer*. Head & neck*. 2011;33(1):65-71. doi: 10.1002/hed.21407.

119. HUTTON JM, WILLIAMS M. An investigation of psychological distress in patients who have been treated for head and neck cancer*. British journal of oral & maxillofacial surgery*. 2001;39(5):333-339. doi: 10.1054/bjom.2001.0645.

120. JENEWEIN J, ZWAHLEN RA, ZWAHLEN D, DRABE N, MOERGELI H, BÜCHI S. Quality of life and dyadic adjustment in oral cancer patients and their female partners*. European journal of cancer care*. 2008;17(2):127-135. doi: 10.1111/j.1365-2354.2007.00817.x.

121. Johansson M, Rydén A, Finizia C. Self evaluation of communication experiences after laryngeal cancer – A longitudinal questionnaire study in patients with laryngeal cancer*. BMC Cancer*. 2008;8(1):80. doi: 10.1186/1471-2407-8-80.

122. Kelly C, Paleri V, Downs C, Shah R. Deterioration in quality of life and depressive symptoms during radiation therapy for head and neck cancer*. Otolaryngology-head and neck surgery*. 2007;136(1):108-111. doi: 10.1016/j.otohns.2006.06.1278.

123. Krebbers I, Simon S, Pilz W, Kremer B, Winkens B, Baijens LJ. Patients with Head-and-Neck Cancer: Dysphagia and Affective Symptoms*. Folia phoniatrica et logopaedica*. 2021;73(4):308-315. doi: 10.1159/000508367.

124. Liu L, Liu D, Guo Q, Shen B. Quality of Life in Advanced Maxillary Sinus Cancer After Radical Versus Conservative Maxillectomy*. The Journal of craniofacial surgery*. 2013;24(4):1368-1372. doi: 10.1097/SCS.0b013e31828601d6.

125. McCarter K, Baker AL, Wolfenden L, et al. Smoking and other health factors in patients with head and neck cancer*. Cancer epidemiology*. 2022;79:102202. doi: 10.1016/j.canep.2022.102202.

126. McDowell LJ, Rock K, Xu W, et al. Long-Term Late Toxicity, Quality of Life, and Emotional Distress in Patients With Nasopharyngeal Carcinoma Treated With Intensity Modulated Radiation Therapy*. International journal of radiation oncology, biology, physics*. 2018;102(2):340-352. doi: 10.1016/j.ijrobp.2018.05.060.

127. Neilson K, Pollard A, Boonzaier A, et al. A longitudinal study of distress (depression and anxiety) up to 18 months after radiotherapy for head and neck cancer*. Psycho-oncology (Chichester, England)*. 2013;22(8):1843-1848. doi: 10.1002/pon.3228.

128. Nikoloudi M, Lymvaios I, Zygogianni A, et al. Quality of life, anxiety, and depression in the head-and-neck cancer patients, undergoing intensity-modulated radiotherapy treatment*. Indian Journal of Palliative Care*. 2020;26(1):54-59. doi: 10.4103/IJPC.IJPC_168_19.

129. Offerman MPJ, Schroevers MJ, van der Velden L, de Boer MF, Pruyn JFA. Goal processes & self-efficacy related to psychological distress in head & neck cancer patients and their partners*. European journal of oncology nursing : the official journal of European Oncology Nursing Society*. 2010;14(3):231-237. doi: 10.1016/j.ejon.2010.01.022.

130. Pandey M, Devi N, Thomas BC, Vinod Kumar S, Krishnan R, Ramdas K. Distress overlaps with anxiety and depression in patients with head and neck cancer*. Psycho-oncology (Chichester, England)*. 2007;16(6):582-586. doi: 10.1002/pon.1123.

131. Pauli N, Johnson J, Finizia C, Andréll P. The incidence of trismus and long-term impact on health-related quality of life in patients with head and neck cancer*. Acta oncologica*. 2013;52(6):1137-1145. doi: 10.3109/0284186X.2012.744466.

132. Petruson KM, Silander EM, Hammerlid EB. Effects of psychosocial intervention on quality of life in patients with head and neck cancer*. Head & neck*. 2003;25(7):576-584. doi: 10.1002/hed.10243.

133. Petruson KM, Silander EM, Hammerlid EB. Quality of life as predictor of weight loss in patients with head and neck cancer*. Head & neck*. 2005;27(4):302-310. doi: 10.1002/hed.20172.

134. Rampling T, King H, Mais KL, et al. Quality of Life Measurement in the Head and Neck Cancer Radiotherapy Clinic: Is it Feasible and Worthwhile?*. Clinical oncology (Royal College of Radiologists (Great Britain))*. 2003;15(4):205-210. doi: 10.1016/S0936-6555(02)00418-1.

135. Rogers SN, Rajlawat B, Goru J, Lowe D, Humphris GM. Comparison of the domains of anxiety and mood of the University of Washington Head and Neck Cancer Questionnaire (UW-QOL V4) with the CES-D and HADS*. Head & neck*. 2006;28(8):697-704. doi: 10.1002/hed.20389.

136. Rose P, Yates P. Quality of Life Experienced by Patients Receiving Radiation Treatment for Cancers of the Head and Neck*. Cancer nursing*. 2001;24(4):255-263. doi: 10.1097/00002820-200108000-00002.

137. Singer S, Danker H, Dietz A, et al. Sexual Problems After Total or Partial Laryngectomy*. The Laryngoscope*. 2008;118(12):2218-2224. doi: 10.1097/MLG.0b013e318182cdc6.

138. So N, McDowell LJ, Lu L, et al. The Prevalence and Determinants of Return to Work in Nasopharyngeal Carcinoma Survivors*. International journal of radiation oncology, biology, physics*. 2020;106(1):134-145. doi: 10.1016/j.ijrobp.2019.09.008.

139. Sunderland M, Matthews C, Waterhouse D, Shetty S, Morton RP. Unmet needs, quality of life and psychological distress: insights regarding head and neck cancer patients in a rural setting*. Journal of laryngology and otology*. 2023;137(1):89-95. doi: 10.1017/S0022215121001699.

140. Suzuki M, Deno M, Myers M, et al. Anxiety and depression in patients after surgery for head and neck cancer in Japan*. Palliative & supportive care*. 2016;14(3):269-277. doi: 10.1017/S1478951515000930.

141. Tang Y, Hua Y, Huang X, Cao Y, Sun X. Psychological Burden of Patients with Head and Neck Cancer Undergoing Radiotherapy and Their Family Caregivers: A Cross-Sectional Survey*. Journal of multidisciplinary healthcare*. 2023;16:927-935. doi: 10.2147/JMDH.S398064.

142. Veer V, Kia S, Papesch M. Anxiety and depression in head and neck out-patients*. Journal of laryngology and otology*. 2010;124(7):774-777. doi: 10.1017/S0022215110000502.

143. Wulff NB, Dalton SO, Wessel I, et al. Health‐Related Quality of Life, Dysphagia, Voice Problems, Depression, and Anxiety After Total Laryngectomy*. The Laryngoscope*. 2022;132(5):980-988. doi: 10.1002/lary.29857.

144. Zahid N, Zahid W, Khalid W, et al. Resilience and its associated factors in head and neck cancer patients in Pakistan: an analytical cross-sectional study*. BMC cancer*. 2021;21(1):1-888. doi: 10.1186/s12885-021-08624-8.

145. Zwahlen, Roger A., MD, DMD, Dannemann C, MD, Grätz, Klaus W., MD, DMD, et al. Quality of Life and Psychiatric Morbidity in Patients Successfully Treated for Oral Cavity Squamous Cell Cancer and Their Wives*. Journal of oral and maxillofacial surgery*. 2008;66(6):1125-1132. doi: 10.1016/j.joms.2007.09.003.

146. Schiefke F, Akdemir M, Weber A, Akdemir D, Singer S, Frerich B. Function, postoperative morbidity, and quality of life after cervical sentinel node biopsy and after selective neck dissection*. Head & neck*. 2009;31(4):503-512. doi: 10.1002/hed.21001.

147. Shiraz F, Rahtz E, Bhui K, Hutchison I, Korszun A. Quality of life, psychological wellbeing and treatment needs of trauma and head and neck cancer patients*. British journal of oral & maxillofacial surgery*. 2014;52(6):513-517. doi: 10.1016/j.bjoms.2014.03.019.

148. Posluszny DM, Dougall AL, Johnson JT, et al. Posttraumatic stress disorder symptoms in newly diagnosed patients with head and neck cancer and their partners*. Head & neck*. 2015;37(9):1282-1289. doi: 10.1002/hed.23760.

149. Ghiggia A, Castelli L, Riva G, et al. Psychological distress and coping in nasopharyngeal cancer: an explorative study in Western Europe*. Psychology, health & medicine*. 2017;22(4):449-461. doi: 10.1080/13548506.2016.1220600.

150. Rodrigues‐Oliveira L, Kauark‐Fontes E, Alves CGB, et al. COVID‐19 impact on anxiety and depression in head and neck cancer patients: A cross‐sectional study*. Oral diseases*. 2022;28(S2):2391-2399. doi: 10.1111/odi.13876.

151. Joseph LA, Routledge JA, Burns MP, et al. Value of the Hospital Anxiety and Depression Scale in the follow up of head and neck cancer patients*. Journal of laryngology and otology*. 2013;127(3):285-294. doi: 10.1017/S0022215113000078.

152. van Nieuwenhuizen AJ, Buffart LM, Smit JH, et al. A comprehensive assessment protocol including patient reported outcomes, physical tests, and biological sampling in newly diagnosed patients with head and neck cancer: is it feasible?*. Support Care Cancer*. 2014;22(12):3321-3330. doi: 10.1007/s00520-014-2359-0.

153. Verdonck-de Leeuw IM, van Bleek W, René Leemans C, de Bree R. Employment and return to work in head and neck cancer survivors*. Oral oncology*. 2010;46(1):56-60. doi: 10.1016/j.oraloncology.2009.11.001.

154. Bozec A, Boscagli M, Serris M, et al. Long-term functional and quality of life outcomes in laryngectomized patients after successful voice restoration using tracheoesophageal prostheses*. Surgical oncology*. 2021;38:101580. doi: 10.1016/j.suronc.2021.101580.

155. Budhrani-Shani P, Chau NG, Berry DL. Psychosocial distress and the preferred method of delivery of mind-body interventions among patients with head-and-neck cancer*. Patient related outcome measures*. 2018;9:129-136. doi: 10.2147/PROM.S149978.

156. Bornbaum CC, Fung K, Franklin JH, Nichols A, Yoo J, Doyle PC. A descriptive analysis of the relationship between quality of life and distress in individuals with head and neck cancer*. Support Care Cancer*. 2012;20(9):2157-2165. doi: 10.1007/s00520-011-1326-2.

157. Bozec A, Majoufre C, De Boutray M, et al. Oral and oropharyngeal cancer surgery with free-flap reconstruction in the elderly: Factors associated with long-term quality of life, patient needs and concerns. A GETTEC cross-sectional study*. Surgical oncology*. 2020;35:81-88. doi: 10.1016/j.suronc.2020.08.014.

158. Chen S, Liao C, Lin C, Chang JT, Lai Y. Distress and care needs in newly diagnosed oral cavity cancer patients receiving surgery*. Oral oncology*. 2009;45(9):815-820. doi: 10.1016/j.oraloncology.2009.01.001.

159. Hong JS, Tian J. Prevalence of anxiety and depression and their risk factors in Chinese cancer patients*. Support Care Cancer*. 2014;22(2):453-459. doi: 10.1007/s00520-013-1997-y.

160. Lazure KE, Lydiatt WM, Denman D, Burke WJ. Association between depression and survival or disease recurrence in patients with head and neck cancer enrolled in a depression prevention trial*. Head & neck*. 2009;31(7):888-892. doi: 10.1002/hed.21046.

161. Riedl D, Gastl R, Gamper E, et al. Cancer patients’ wish for psychological support during outpatient radiation therapy*. Strahlenther Onkol*. 2018;194(7):655-663. doi: 10.1007/s00066-018-1288-0.

162. Laurence B, Mould‐Millman N, Nero KE, Salter RO, Sagoo PK. Depression and hospital admission in older patients with head and neck cancer: analysis of a national healthcare database*. Gerodontology*. 2017;34(2):284-287. doi: 10.1111/ger.12247.

163. Li S, Lee YA, Li Q, et al. Oral lesions, chronic diseases and the risk of head and neck cancer*. Oral oncology*. 2015;51(12):1082-1087. doi: 10.1016/j.oraloncology.2015.10.014.

164. Rieke K, Boilesen E, Lydiatt W, Schmid KK, Houfek J, Watanabe-Galloway S. Population-based retrospective study to investigate preexisting and new depression diagnosis among head and neck cancer patients*. Cancer epidemiology*. 2016;43:42-48. doi: 10.1016/j.canep.2016.06.008.

165. Rohde RL, Adjei Boakye E, Challapalli SD, et al. Prevalence and sociodemographic factors associated with depression among hospitalized patients with head and neck cancer—Results from a national study*. Psycho-oncology (Chichester, England)*. 2018;27(12):2809-2814. doi: 10.1002/pon.4893.

166. Rieke K, Schmid KK, Lydiatt W, Houfek J, Boilesen E, Watanabe-Galloway S. Depression and survival in head and neck cancer patients*. Oral oncology*. 2017;65:76-82. doi: 10.1016/j.oraloncology.2016.12.014.

167. Bigelow EO, Blackford AL, Eytan DF, Eisele DW, Fakhry C. Burden of comorbidities is higher among elderly survivors of oropharyngeal cancer compared with controls*. Cancer*. 2020;126(8):1793-1803. doi: 10.1002/cncr.32703.

168. Jeffery DD, Art Ambrosio L, Hopkins L, Burke HB. Mental health comorbidities and cost/utilization outcomes in head and neck cancer patients*. Journal of psychosocial oncology*. 2019;37(3):301-318. doi: 10.1080/07347332.2018.1519626.

169. Madrigal J, Tie EK, Verma A, Benharash P, Rapkin DA, St John MA. The Increasing Burden of Depression in Patients Undergoing Head and Neck Cancer Operations*. The Laryngoscope*. 2023. doi: 10.1002/lary.30735.

170. Mirosevic S, Thewes B, van Herpen C, et al. Prevalence and clinical and psychological correlates of high fear of cancer recurrence in patients newly diagnosed with head and neck cancer*. Head & Neck*. 2019;41(9):3187-3200. doi: 10.1002/hed.25812.

171. Mukherjee A, Bhowmick C, Chattopadhyay S, et al. Preoperative risk factors associated with peri-operative psychiatric diagnosis in oral cancer patients*. Ecancermedicalscience*. 2022;16:1401. doi: 10.3332/ecancer.2022.1401.

172. Wang C, Chen J, Su L, et al. The psychological status in patients with nasopharyngeal carcinoma during radiotherapy*. Eur Arch Otorhinolaryngol*. 2022;279(2):1035-1042. doi: 10.1007/s00405-021-06892-5.

173. Choi JW, Park E. Suicide risk after cancer diagnosis among older adults: A nationwide retrospective cohort study*. Journal of geriatric oncology*. 2020;11(5):814-819. doi: 10.1016/j.jgo.2019.11.006.

174. Osazuwa-Peters N, Arnold LD, Loux TM, Varvares MA, Schootman M. Factors associated with increased risk of suicide among survivors of head and neck cancer: A population-based analysis*. Oral oncology*. 2018;81:29-34. doi: 10.1016/j.oraloncology.2018.03.017.

175. Nugent SM, Morasco BJ, Handley R, et al. Risk of Suicidal Self-directed Violence Among US Veteran Survivors of Head and Neck Cancer*. Archives of otolaryngology--head & neck surgery*. 2021;147(11):981-989. doi: 10.1001/jamaoto.2021.2625.

176. SAVARD J, VILLA J, IVERS H, SIMARD S, MORIN CM. Prevalence, Natural Course, and Risk Factors of Insomnia Comorbid With Cancer Over a 2-Month Period*. Journal of clinical oncology*. 2009;27(31):5233-5239. doi: 10.1200/JCO.2008.21.6333.

177. Rapoport Y, Kreitler S, Chaitchik S, Algor R, Weissler K. Psychosocial problems in head-and-neck cancer patients and their change with time since diagnosis*. Annals of oncology*. 1993;4(1):69-73. doi: 10.1093/oxfordjournals.annonc.a058365.

178. Hajdú SF, Wessel I, Dalton SO, Eskildsen SJ, Johansen C. Swallowing Exercise During Head and Neck Cancer Treatment: Results of a Randomized Trial*. Dysphagia*. 2022;37(4):749-762. doi: 10.1007/s00455-021-10320-5.

179. FUJII M, OHNO Y, TOKUMARU Y, et al. Manifest Anxiety Scale for evaluation of effects of granisetron in chemotherapy with CDDP and 5FU for head and neck cancer*. Supportive care in cancer*. 2001;9(5):366-371. doi: 10.1007/s005200000221.

180. Yadav P, Karkal R, Kakunje A, Mahatme N, Akhilesh M. Prevalence of depressive disorders among head-and-neck cancer patients: A hospital-based, cross-sectional study*. Indian Journal of Psychiatry*. 2019;61(4):409-414. doi: 10.4103/psychiatry.IndianJPsychiatry_511_18.

181. Moschopoulou E, Hutchison I, Bhui K, Korszun A. Post-traumatic stress in head and neck cancer survivors and their partners*. Support Care Cancer*. 2018;26(9):3003-3011. doi: 10.1007/s00520-018-4146-9.

182. Britton B, Baker AL, Wolfenden L, et al. Eating As Treatment (EAT): A Stepped-Wedge, Randomized Controlled Trial of a Health Behavior Change Intervention Provided by Dietitians to Improve Nutrition in Patients With Head and Neck Cancer Undergoing Radiation Therapy (TROG 12.03)*. International journal of radiation oncology, biology, physics*. 2019;103(2):353-362. doi: 10.1016/j.ijrobp.2018.09.027.

183. Hammermüller C, Hinz A, Dietz A, et al. Depression, anxiety, fatigue, and quality of life in a large sample of patients suffering from head and neck cancer in comparison with the general population*. BMC cancer*. 2021;21(1):94. doi: 10.1186/s12885-020-07773-6.

184. Manne SL, Hudson SV, Kashy DA, et al. Self‐efficacy in managing post‐treatment care among oral and oropharyngeal cancer survivors*. European journal of cancer care*. 2022;31(6):e13710-n/a. doi: 10.1111/ecc.13710.

185. McCarter K, Baker AL, Britton B, et al. Smoking, drinking, and depression: comorbidity in head and neck cancer patients undergoing radiotherapy*. Cancer Medicine*. 2018;7(6):2382-2390. doi: 10.1002/cam4.1497.

186. Omoro SAO, Fann JR, Weymuller EA, MacHaria IM, Yueh B. Swahili Translation and Validation of the Patient Health Questionnaire-9 Depression Scale in the Kenyan Head and Neck Cancer Patient Population*. International journal of psychiatry in medicine*. 2006;36(3):367-381. doi: 10.2190/8W7Y-0TPM-JVGV-QW6M.

187. Joseph N, Prakash Saxena P, Shettigar A, Kotian S. Assessment of fatigability, depression, and self-esteem among head-and-neck carcinoma patients in a tertiary care hospital in South India*. Journal of Cancer Research and Therapeutics*. 2019;15(3):645-652. doi: 10.4103/jcrt.JCRT_277_17.

188. Shinn EH, Valentine A, Jethanandani A, et al. Depression and Oropharynx Cancer Outcome*. Psychosomatic medicine*. 2016;78(1):38-48. doi: 10.1097/PSY.0000000000000256.

189. Torrealba MNR, das Mercês NNA, Felix JVC, Paes MR, Pereira DKV, Sartor SF. Hope and depression in Brazilian head and neck cancer patients during the COVID-19 pandemic*. Ecancermedicalscience*. 2022;16:1371. doi: 10.3332/ecancer.2022.1371.

190. Eastburn K, Lyu L, Harrison C, et al. Association Between Patient-Reported Symptoms of Dysphagia and Psychological Distress in Head and Neck Cancer Survivors*. Oncology Nursing Forum*. 2022;49(1):81-89A. doi: 10.1188/22.ONF.81-89.

191. Gascon B, Panjwani AA, Mazzurco O, Li M. Screening for Distress and Health Outcomes in Head and Neck Cancer*. Current oncology (Toronto)*. 2022;29(6):3793-3806. doi: 10.3390/curroncol29060304.

192. Macias D, Hand BN, Pipkorn P, et al. Association of Inventory to Measure and Assess imaGe Disturbance – Head and Neck Scores With Clinically Meaningful Body Image-Related Distress Among Head and Neck Cancer Survivors*. Frontiers in psychology*. 2021;12:794038. doi: 10.3389/fpsyg.2021.794038.

193. Li N, Otomaru T, Taniguchi H. Sleep quality in long-term survivors of head and neck cancer: preliminary findings*. Support Care Cancer*. 2017;25(12):3741-3748. doi: 10.1007/s00520-017-3804-7.

194. Santoso AMM, Jansen F, Lissenberg-Witte BI, et al. Poor sleep quality among newly diagnosed head and neck cancer patients: prevalence and associated factors*. Support Care Cancer*. 2021;29(2):1035-1045. doi: 10.1007/s00520-020-05577-9.

195. Richardson AE, Morton RP, Broadbent E. Coping strategies predict post-traumatic stress in patients with head and neck cancer*. Eur Arch Otorhinolaryngol*. 2016;273(10):3385-3391. doi: 10.1007/s00405-016-3960-2.

196. Barber B, Dergousoff J, Nesbitt M, et al. Depression as a predictor of postoperative functional performance status (PFPS) and treatment adherence in head and neck cancer patients: a prospective study*. Journal of Otolaryngology - Head & Neck Surgery*. 2015;44(1):38. doi: 10.1186/s40463-015-0092-4.

197. Katz MR, Kopek N, Waldron J, Devins GM, Tomlinson G. Screening for depression in head and neck cancer*. Psycho-oncology (Chichester, England)*. 2004;13(4):269-280. doi: 10.1002/pon.734.

198. Sehlen S, Lenk M, Herschbach P, et al. Depressive symptoms during and after radiotherapy for head and neck cancer*. Head & neck*. 2003;25(12):1004-1018. doi: 10.1002/hed.10336.

199. Liu Q, Wang X, Kong X, et al. Subsequent risk of suicide among 9,300,812 cancer survivors in US: A population-based cohort study covering 40 years of data*. EClinicalMedicine*. 2022;44:101295. doi: 10.1016/j.eclinm.2022.101295.

200. Lou S, Xu D, Li X, Huan Y, Li J. Study of psychological state of cancer patients undergoing radiation therapy during novel coronavirus outbreak and effects of nursing intervention*. Precision Medical Sciences*. 2020;9(2):83-89. doi: 10.1002/prm2.12026.

201. Firmeza MA, Rodrigues AB, Melo GAA, et al. Control of anxiety through music in a head and neckoutpatient clinic: a randomized clinical trial*. Revista da Escola de Enfermagem da U S P*. 2017;51:e03201. doi: 10.1590/s1980-220x2016030503201.

202. Chang Y, Huang P, Liao C, Wang H, Lin C, Chen S. Factors impacting posttraumatic growth in head-and-neck cancer patients with oncologic emergencies*. Support Care Cancer*. 2022;30(5):4515-4525. doi: 10.1007/s00520-021-06772-y.

203. Jehn P, Linsen SS, Zeller A, et al. Gender-specific differences concerning psychosocial aspects and functional impairments that influence quality of life in oral cancer treatment*. Support Care Cancer*. 2022;30(6):4905-4915. doi: 10.1007/s00520-022-06907-9.

204. Chang D, Chen AW, Lo Y, Chuang Y, Chen M. Factors associated with suicidal ideation risk in head and neck cancer: A longitudinal study*. The Laryngoscope*. 2019;129(11):2491-2495. doi: 10.1002/lary.27843.

205. Tseng W, Lee Y, Hung C, et al. Stigma, depression, and anxiety among patients with head and neck cancer*. Support Care Cancer*. 2022;30(2):1529-1537. doi: 10.1007/s00520-021-06550-w.

206. Hadas S, Huhn M, Rentrop M, et al. The role of psycho-oncologic screenings in the detection and evaluation of depression in head and neck cancer aftercare patients*. Eur Arch Otorhinolaryngol*. 2022;279(4):2143-2156. doi: 10.1007/s00405-021-07017-8.
